# Efficacy of Chlorogenic Acid in Treating Tripterygium Glycoside-Induced Asthenozoospermia in Rats and Its Possible Mechanisms

**DOI:** 10.3390/vetsci12010066

**Published:** 2025-01-16

**Authors:** Long Chen, God’spower Bello-Onaghise, Mo Chen, Shunda Li, Yu Zhang, Haoran Wang, Qianwei Qu, Yanhua Li

**Affiliations:** 1 College of Veterinary Medicine, Northeast Agricultural University, 600 Changjiang Road, Xiangfang, Harbin 150030, China; s220602074@neau.edu.cn (L.C.); godspower.bello-onaghise@uniben.edu (G.B.-O.); s220602070@neau.edu.cn (S.L.); s230602064@neau.edu.cn (Y.Z.); qwqu@neau.edu.cn (Q.Q.); 2 Department of Animal Science, Faculty of Agriculture, University of Benin, Benin City 300103, Nigeria; 3 State Key Laboratory of Veterinary Biotechnology, Harbin Veterinary Research Institute, Chinese Academy of Agricultural Sciences, Harbin 150069, China; chenmo1212@foxmail.com; 4 Department of Clinical Medicine, School of Clinical Medicine, Southern Medical University, 1023 Shatainan Road, Guangzhou 510515, China; 3200901024@i.smu.edu.cn

**Keywords:** Tripterygium glucosides, asthenozoospermia, chlorogenic acid, antioxidants, oxidative stress, network pharmacology

## Abstract

Tripterygium glycoside (TG) is a potent medication for treating autoimmune diseases. However, it has also been associated with toxic effects on testicular tissues. This study evaluated the antioxidant potential of chlorogenic acid (CGA) in protecting testicular tissues in rats with TG-induced asthenozoospermia (AZS). A total of 45 6-week-old Sprague–Dawley rats were divided into a control (10 rats) and a TG group (35 rats). The control group received 10 mL of deionized water, while the TG group received 40 mg/kg TG/i.g./28 d to establish the asthenozoospermia (AZS) model as assessed by sacrificing five rats from each group. The remaining 5 and 30 rats were divided into control, TG + LC, TG, TG + CGA-L, TG + CGA-M, TG + CGA-H groups, *n* = 5. The control and TG groups received 10 mL of deionized water. TG + LC received L-carnitine (0.2 g/kg/d), and the TG + CGA-L, TG + CGA-M, and TG + CGA-H groups received 25 mg/kg, 50 mg/kg, and 100 mg/kg of CGA, respectively, via gavage for 28 days. From the results, TG effectively induced asthenozoospermia in the rats through oxidative damage to testicular tissues, significantly increasing ROS and MDA along with hormonal and physio-biochemical dysfunction. Simultaneously, CGA treatment ameliorated the toxicity of TG.

## Simple Summary

Tripterygium glycosides (TGs), derived from the traditional Chinese medicine Tripterygium wilfordii, are primarily utilized for treating autoimmune diseases like rheumatoid arthritis. However, TG is associated with serious side effects, such as liver damage, heart toxicity, and reproductive issues, which limit its clinical use. Clinical studies indicate that around 23% of TG users experience adverse reactions, particularly reproductive complications, with men being more affected. Conditions like asthenozoospermia, characterized by low sperm counts, are prevalent among infertile men and are often linked to oxidative stress from elevated levels of reactive oxygen species (ROS). This oxidative stress can damage sperm DNA and membranes, contributing to infertility. Antioxidants may help mitigate these effects, and one promising candidate is chlorogenic acid (CGA)—a compound found in various plants used in traditional Chinese medicine. CGA exhibits significant health benefits, including antioxidant properties. This study established an asthenozoospermia (AZS) model in male rats through the intragastric administration of TGs and explored the antioxidant potential of CGA in alleviating the oxidative stress-induced asthenozoospermia condition. With the help of the network pharmacology approach, we identified the MAPK pathway as the signaling pathway implicated in the elevation of oxidative stress markers in the TG-induced asthenozoospermia model group of rats. The same pathway was also used to elucidate the mechanisms by which CGA counteracted the reproductive toxicity of TG, ultimately highlighting its protective role in sperm health.

## 1. Introduction

Tripterygium glycosides (TGs) are the most common form of traditional Chinese medicine, known as Tripterygium wilfordii Hook F (TWHF). While TG has shown significant efficacy in treating systemic lupus erythematosus and various autoimmune conditions, particularly rheumatoid arthritis (RA) [1,2,3,4], its notable toxicity must not be overlooked. Severe instances of liver damage, heart toxicity, reproductive issues, and damage to multiple organs have been frequently documented, significantly limiting its practical use in clinical settings [5,6,7]. Recent studies have suggested that the plant’s reproductive toxicity may lead to organ shrinkage, reduced function, and cell death. However, the exact molecular mechanisms behind these effects are not fully understood due to the complex nature of the plant’s composition and the impact of the elevated levels of reactive oxygen species (ROS) it generates. A clinical meta-analysis focusing on the use of TG in rheumatoid arthritis (RA) patients revealed an overall adverse reaction rate of 23%, with reproductive system complications being the most prevalent at 14% [8,9]. Women may experience amenorrhea, while men might encounter reduced sperm motility and potential infertility [10,11,12,13]. Notably, the male population appears to be more vulnerable to the reproductive toxicity triggered by TG than females, showing higher incidence rates and a faster onset of symptoms [14,15].

Irregularities in sperm count, a common characteristic of asthenozoospermia, have been identified as the most common abnormality and are usually a significant contributing factor in men diagnosed as infertile [16,17]. Abnormalities in semen parameters can arise from various factors like age, chromosomal anomalies, hormonal imbalances, or infections and are frequently of unknown origin [18]. Researchers have sought to explore the connection between male infertility, sperm quality, and abnormalities in semen parameters. Previous studies have also investigated the links between reactive oxygen species (ROS) and male infertility [19,20,21]. Reactive oxygen species (ROS) are important players in cellular proliferation, differentiation, migration, apoptosis, and necrosis. Low to intermediate levels of ROS and RNS are necessary to maintain many important physiological functions, redox homeostasis, and the regulation of key transcription factors. In contrast, the excessive formation of ROS is responsible for disrupted redox homeostasis, which in turn leads to oxidative stress and ROS-mediated damage to all important biomolecules, including DNA, proteins, and membranes [22]. Oxidative stress, characterized by a shifted equilibrium between the formation and elimination of free radicals toward the formation, is a common denominator of the pathogenesis of chronic diseases and aging [23]. Oxidative stress refers to a state where the levels of oxygen and oxygen-derived free radicals exceed the cell’s natural antioxidant defenses [24]. This condition negatively impacts sperm function by damaging the integrity of DNA, which occurs alongside harm to the proteins and lipids in the sperm cell plasma membrane, thereby disrupting cell membrane fluidity and permeability [25]. DNA fragmentation is most commonly observed in the spermatozoa of infertile men, with substantial evidence indicating that this damage is free radical-mediated [26,27,28,29]. Spermatozoa with extensive DNA damage will encounter biological and molecular barriers that likely preclude fertilization, such as a lack of viable DNA, which ultimately prevents the transmission of damaged DNA during fertilization. Although these stages are bypassed by the use of assisted reproductive technologies (ARTs), they may have adverse effects on the health of offspring, potentially doubling the incidence of infertility and increasing the rates of several childhood and genetic disorders [30,31]. Furthermore, sperm mitochondria serve as both the source and often the targets of free-radical oxidation. Mitochondrial DNA is particularly susceptible to damage since it lacks the protection of histones and has a limited capacity for DNA repair due to the absence of nucleotide-excision repair pathways [32]. Consequently, the mutation rate of mitochondrial DNA is estimated to be two orders of magnitude higher than that of nuclear DNA. Sperm cells with a high number of damaged mitochondria are unable to undergo apoptosis due to this damage, leading to the presence of sperm with compromised DNA in the ejaculate [33]. Dysfunctional mitochondria carrying mutations generate more free radicals and produce less ATP than their fully functional counterparts. The accumulation of a large number of such mitochondria may result in hypospermatogenesis, which can stem from meiotic arrest during sperm cell development or the presence of a partially formed and disorganized axonemal apparatus, ultimately leading to asthenozoospermia [34]. In a separate study, Aitken et al. [35] found that oxidative damage to the lipids in the sperm plasma membrane due to the accumulation of ROS could be reversed by the scavenging properties of antioxidants. They do this by effectively disrupting the peroxidation process.

This suggests a potential way to protect sperm health and improve fertility. Chlorogenic acid (CGA), a depside acid, is produced by combining caffeic acid and quinic acid (1-hydroxyhexahydrogallic acid). It is a phenylacrylate polyphenol compound created through the shikimic acid pathway in plants during aerobic respiration. Its hemihydrate forms white or slightly yellow needle-like crystals, which are poorly soluble in organic solvents like chloroform, ether, and benzene but readily dissolve in polar solvents such as methanol, ethanol, and acetone. CGA is found in a variety of sources in traditional Chinese medicine, including honeysuckle [36], potato [37], cork [38], Eucommia leaves [39], chrysanthemum [40], strawberry [41], mango [42], blueberries [43], mulberry leaves [44], and green coffee [45]. Researchers have recently utilized high-voltage discharges to extract CGA from different parts of tobacco waste (waste, dust, and midrib) [46]. Studies conducted in the laboratory and on animals have demonstrated that chlorogenic acid exhibits several important biological activities, such as antioxidant properties, liver and kidney protection, antibacterial effects, anti-tumor activity, regulation of sugar and lipid metabolism, anti-inflammatory properties, and safeguarding the nervous system [47].

Network pharmacology is a multifaceted field that combines systematic biology theory [48], pharmacology, information networking, and computer science. This concept encompasses the theory of holism, which is the basic principle of traditional Chinese medicine (TCM) [6]. By utilizing computer simulations and numerous databases, network pharmacology screens drug molecular targets and disease-related targets. Through high-throughput screening, network visualization, and network analysis, it uncovers the intricate connections between drugs, targets, and diseases, ultimately analyzing and predicting the mechanisms of drugs [49]. Additionally, if needed, it further verifies the effects of drugs through corresponding experiments [50] and predicts their possible mechanisms.

In this study, we created a model of asthenozoospermia in male rats using Tripterygium glycosides (TG) and explored the efficacy of chlorogenic acid (CGA) in ameliorating this condition. We utilized network pharmacology techniques to uncover the potential mechanisms through which chlorogenic acid attenuated TG-induced asthenozoospermia.

## 2. Materials and Methods

### 2.1. Experimental Animals

A total of 45 6-week-old Sprague–Dawley rats, weighing 200 ± 50 g, were purchased from Liaoning Changsheng Biotechnology Co., Ltd. and randomly divided into separate cages. They were housed under standard conditions of temperature (22 ± 3 °C), relative humidity (55 ± 5%), and a light/dark cycle (12 h of light/12 h of dark) throughout this study. The rats were fed a standard pellet-chow diet and given access to water ad libitum. Before the commencement of the trials, all animals were allowed to acclimatize to the laboratory conditions for 1 week. All animal experiments were conducted following the ARRIVE guidelines [51]. After the one-week adaptation period, the rats were randomly assigned to two groups: a control group and a model group. The control group consisted of 10 rats, while the model group included 35. The model group received a daily dose of 40 mg/kg of Tripterygium wilfordii polyglycoside (TG) tablet suspensions for 28 days. The control group was administered 10 mL/kg of deionized water via gavage daily for the same period.

At the end of this phase, five rats from each group were randomly selected, weighed, and euthanized. One epididymis was collected from each rat for routine semen analysis, and blood samples were gathered for serum sex hormone level testing to confirm the establishment of the asthenozoospermia model. The remaining five rats in the control group were maintained as controls. The remaining 30 rats in the TG group were randomly divided into five subgroups: the TG group (TG), the TG + L-carnitine control group (TG + LC), the low-dose chlorogenic acid group (TG + CGA-L), the medium-dose chlorogenic acid group (TG + CGA-M), and the high-dose chlorogenic acid group (TG + CGA-H), with five rats in each subgroup (n = 5). The procedures involved in this study were approved and registered by the Northeast Agricultural University Institutional Animal Care and Use Committee, Harbin, China (NEAUEC2024 03 125).

### 2.2. Tripterygium Wilfordii Glycosides (TGs) Dosage Preparation and Administration

Commercially available Tripterygium wilfordii polyglycoside tablets, containing 10 mg of Tripterygium glycoside per tablet, were purchased from Yuanda Pharmaceutical Huangshi Feiyun Pharmaceutical Co., Ltd, (Batch No.: 20230801, Huangshi, China). The tablets were homogenized, and a suspension was prepared at a concentration of 5 mg/mL to achieve a dosage of 40 mg/kg body weight per day using a saline solution. The concentration was adjusted weekly to maintain the dosage as the rats’ body weights increased. A 2 mL/kg b.wt. aliquot of the suspension was administered to the rats daily, with continuous weekly adjustments for 4 weeks via gavage, immediately after preparation. The dosage of the TG tablet solution was maintained at 40 mg/kg throughout this study. To ensure accuracy, we calculated the dosage based on the rats’ weight at each time point and confirmed that the dosage was consistently maintained at 40 mg/kg b.wt throughout this study, with adjustments made to the volume administered to account for the rats’ changing weight. This approach ensured the accuracy and reliability of our study. The dosage of TG tablets used in this study was in line with the range of dosages provided by [52,53,54].

### 2.3. Chlorogenic Acid (CGA) and Levo-Carnitine (L-C) Dosage Preparation and Administration

Chlorogenic acid (purchased from Dalian Meilun Biotechnology Co., Ltd., Batch No.: S0911D, Dalian, China) and L-carnitine (purchased as a commercially available suspension from Northeast Pharmaceutical Group Shenyang First Pharmaceutical Co., Ltd., Batch No.: 231004, Shenyang, China.) were utilized in this experiment. Rats were grouped and weighed, with results recorded weekly. The control group (control) and the model group (TG) received 2 mL/kg b.wt. of deionized water via gavage for 28 days. In contrast, the TG + LC group was administered an L-carnitine suspension at 0.2 g/kg b.wt. by gavage for the same duration. Chlorogenic acid treatment was divided into three dosage groups: the CGA-Low group received 25 mg/kg b.wt., the CGA-Mid group received 50 mg/kg b.wt., and the CGA-High group was given 100 mg/kg b.wt., all in diluted solution via gavage for 28 days. All administered volumes were 2 mL/kg b.wt. per day.

### 2.4. Blood and Tissue Sample Collection and Sample Processing

Before taking blood and tissue samples, the rats were fasted for 12 h and then weighed. They were anesthetized with an intraperitoneal injection of 1 mL/100 g body weight of 10% chloral hydrate, and blood samples were collected from the abdominal aorta for the serum analysis of sex hormones and biochemical markers. Serum sex hormones, including testosterone (T), luteinizing hormone (LH), and follicle-stimulating hormone (FSH), were assessed. Additionally, liver function was evaluated by measuring serum levels of aspartate transaminase (AST) and alanine transaminase (ALT), while kidney function was assessed by measuring creatinine (CRE) and blood urea nitrogen (BUN). The left epididymal tail was excised to assess sperm quality, including motility and concentration. Furthermore, the left testis and epididymis were removed to evaluate the antioxidant status of testicular tissue by measuring malondialdehyde (MDA), reactive oxygen species (ROS), superoxide dismutase (SOD), and total antioxidant capacity (T-AOC).

### 2.5. Sperm Quality Analysis

The separated epididymal tail was placed in a 5 mL EP tube along with 3 mL of physiological saline that had been preheated to 37 °C. To facilitate the complete release of sperm, ophthalmic scissors were used to make several incisions in the epididymal tail. The tube was then incubated in a water bath at 37 °C for 5 minutes. After incubation, 10 μL of the suspension was placed on a slide for the automatic assessment of sperm concentration and motility using a sperm quality detection system.

### 2.6. Serum Sex Hormone Levels Measurement

After blood was collected from the abdominal aorta, it was allowed to stand at room temperature for 2 h. Blood samples were then centrifuged at 1000 rpm for 20 min, and the supernatants were collected for the measurement of serum T, LH, and FSH levels. The measurement was carried out strictly according to the instructions provided with each sample kit, and measurement was undertaken at a wavelength of 450 nm.

### 2.7. Measurement of Oxidative Stress Levels in Testicular Tissue

The left testicular tissues were obtained, cut into pieces, and then ground into a homogenate. Reactive oxygen species (ROS), malondialdehyde (MDA), total antioxidant capacity (T-AOC), and superoxide dismutase (SOD) levels in the testicular tissue were detected according to the procedures provided with each kit, following the manufacturer’s instructions.

### 2.8. Measurement of Biochemical and Physiological Parameters

Blood serum was used to determine the levels of serum AST, ALT, BUN, and CRE. The measurements were performed strictly according to the instructions provided with each assay kit.

### 2.9. Possible Mechanisms

Network pharmacology was used to explore the potential mechanisms involved in the development of asthenozoospermia in rats. This process followed the methodology outlined by Ding et al. [55] with some modifications. A comprehensive search of keywords like oligospermia, azoospermia, asthenospermia, asthenozoospermia, sperm disorder, and the names of some bioactive compounds, including chlorogenic acid (CGA), coumestrol (COU), Schisandrin A (SCH A), Schisandrin B (SCH B), Quercetin (QUE), and Gingerol (GIN) were inputted into PubMed (https://pubmed.ncbi.nlm.nih.gov accessed on the 2 October 2024) for the curation of related articles. These articles were screened, and only those related to the bioactive compounds were saved in an Excel sheet.

We searched the TCM system pharmacopeia database (TCMSP) (https://old.tcmsp-e.com/load_intro.php?id=27 accessed on the 2 of October 2024) for detailed chemical information on the compounds, including the compound’s chemical name., molecular weight, ADME parameters [oral bioavailability (OB), drug-likeness (DL), and intestinal epithelial permeability (Caco-2)], CAS ID, ingredient-related targets, diseases, etc.

According to the suggestions from the TCMSP, an OB ≥ 30%, DL index ≥ 0.18, and Caco-2 ≥ −0.4 were chosen as the criteria for screening the bioactive components of TCM (Huang et al., 2014).

The bioactive compounds obtained from TCMSP were validated by PubChem (https://pubchem.ncbi.nlm.nih.gov, accessed on the 2 October 2024), and their 2D structures were obtained and transferred to the SWISSADME database for drug-likeness prediction in line with Lipinski’s rule of five. Generally, Lipinski’s rule of five is a simple approach to assessing the drug-likeness of a compound, even though there are other complex criteria involved.

In this study, we considered compliance with the Lipinski rule of five and the oxygen radical absorbance capacity (ORAC) of the compounds, their availability, and cost. Putting these criteria together, chlorogenic acid was selected. It met the TCMSP and Lipinski’s rule of five criteria. It was comparatively the cheapest among compounds with high ORAC. Its structure was uploaded to the Swiss target prediction database (http://swisstargetprediction.ch, accessed on the 2 October 2024) for bioactive hits, and these hits (gene names) were validated by the version 2024_06 (https://www.uniprot.org accessed on the 2 October 2024). Validated bioactive compound targets were saved in an Excel sheet for further analysis.

The STRING database (version 11, https://string-db.org/ accessed on the 2 October 2024) was utilized to construct the protein–protein interaction (PPI) network, focusing on protein interactions related to target proteins in the Homo sapiens prompt of the STRING database.

This comprehensive network provided insights into all proteins associated with CGA-AZS disorders. A total of 322 targets were identified from sperm disorders, with 92 targets associated with chlorogenic acid (CGA) and other bioactive compounds. Using Venny 2.1 for analysis, we found nine overlapping targets implicated in the sperm asthenozoospermia and CGA interaction, which included AR, ESR1, HIF1A, MAPK1, NFE2L2, PTGS2, TNF, TP53, and GMNN—essential for reproductive health, immune response, inflammation, and cancer prevention.

The common genes were then uploaded to Cytoscape 3.10.1, where hub genes were identified using the degree ranking method via the CytoHubba plugin. This ranked the hub genes that target CGA and AZS (see Table S1). The databases used for the curation of these targets are detailed in Table S1A,B. Protein IDs were validated against the UniProt database (https://www.uniprot.org), and results were compiled in an Excel file for further analysis.

After identifying the key genes within the disease network, we carried out Gene Ontology (GO) and Kyoto Encyclopedia of Genes and Genomes (KEGG) enrichment analyses using GO [56] (http://www.geneontology.org/) to map the signaling pathways involved in the Asthenozoospermia model. The analysis encompassed the exploration of biological processes (BP), cellular components (CC), and molecular functions (MF) through the GO enrichment analysis and pathways involved in the disease pathways.

### 2.10. Western Blotting Analysis

Total proteins were extracted from rat testicular tissues using a cell lysis buffer (92590, Merck Millipore, USA) containing 1 mM phenylmethylsulfonyl fluoride (PMSF) (KGP610, KeyGEN, China). Protein concentration was quantified using the BCA Protein Assay Kit (MK164230, Thermo, Waltham, MA, USA). The proteins were then separated by sodium dodecyl sulfate-polyacrylamide gel electrophoresis (SDS-PAGE) and transferred to polyvinylidene fluoride (PVDF) membranes (ISEQ00010, Merck Millipore, Berlington, MA, USA) using the wet electrophoretic transfer method. The membranes were blocked with 5% nonfat milk for 1 h at room temperature and subsequently incubated overnight at 4 °C with primary antibodies against AKT1 (1:1000, YM3618, Immunoway, Plano, TX, USA) and PI3K (1:1000, YM3503, Immunoway, Plano, TX, USA). The anti-β-actin antibody (1:10,000, bs-0061R, Bioss Biotechnology Co., Ltd., Shanghai, China) was used for normalization. After primary antibody incubation, the membranes were treated with HRP-conjugated Affinipure Goat Anti-Rabbit IgG (H+L) (SA00001-2, 1:5000, Proteintech, Wuhan, China) and HRP-conjugated Affinipure Goat Anti-Mouse IgG (H+L) (SA00001-1, 1:5000, Proteintech, Wuhan, China) for 2 h at room temperature. The protein bands were visualized using an enhanced chemiluminescence detection kit (Pierce, Appleton, WI, USA) and analyzed with a Tanon 5200 chemiluminescence detection system (Tanon, Shanghai, China). Finally, band intensity was quantified using a computer-assisted imaging analysis system (ImageJ, version 1.52, NIH, Bethesda, MD, USA).

### 2.11. Intra-Testicular Sex Hormone Measurement

To measure the levels of intratesticular sex hormones, including testosterone (T), follicle-stimulating hormone (FSH), and luteinizing hormone (LH), the following procedure was performed. First, a testicular tissue sample weighing 0.2 g was obtained. The tissue was then combined with 0.5 mL of phosphate-buffered saline (PBS) in a grinding pestle. The mixture was thoroughly homogenized to ensure a uniform suspension. Following homogenization, the sample was subjected to centrifugation at 3000 rpm for 10 min. to separate the supernatant from any solid debris. The concentration of protein in the supernatant was quantified using a BCA Protein Assay Kit (MK164230, Thermo, Waltham, MA, USA), ensuring accurate measurements for subsequent analyses. To determine the levels of the sex hormones, specific detection kits were employed. Testosterone levels were measured using the T kit (YJ002868, mlbio, Shanghai, China), while the concentrations of FSH and LH were assessed using their respective kits (FSH kit: YJ002872, mlbio, Shanghai, China; LH kit: YJ002860, mlbio, Shanghai, China). Each assay was conducted according to the manufacturer’s instructions to ensure reliable results.

### 2.12. Data Analysis

The data obtained were analyzed using a one-way analysis of variance (ANOVA) with GraphPad Prism 9.5.1. Multiple mean comparisons were performed using Tukey’s Honestly Significant Difference (HSD) test. Results are presented as mean ± standard deviation (X ± SD). The critical significance levels were set at *p* < 0.05 (indicating a significant difference) and *p* < 0.01 (indicating a highly significant difference).

## 3. Results

### 3.1. CGA Treatment Improves Bodyweight Measurement

**Figure 1:**
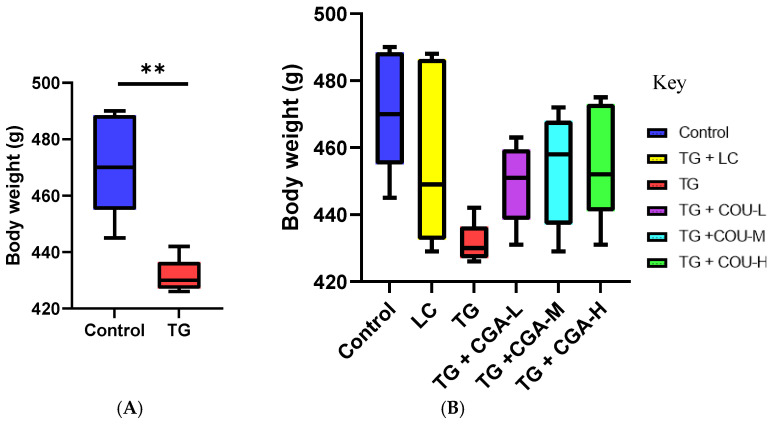
Efficacy of chlorogenic acid (CGA) treatment on the body weights of rats after 28 days of experimentation. (**A**) TG significantly reduced the body weight of rats (** *p* < 0.05). (**B**) Chlorogenic acid progressively increased the body weight of rats after TG-induced reduction. The effect of chlorogenic acid was impressive but not significantly different from TG, same as the LC treatment (*p* > 0.05).

The intragastric administration of 40 mg/kg/d of Tripterygium glycoside (TG) tablets significantly reduced the body weight of rats after 28 days compared to the control (Figure 1A). Conversely, chlorogenic acid (CGA) demonstrated potential in improving the body weights of treated rats. However, the effect of CGA on body weight was not significantly different from the effect of TG in the model group during the period of experimentation (Figure 1B).

### 3.2. CGA Treatment Improves Sperm Quality

**Figure 2:**
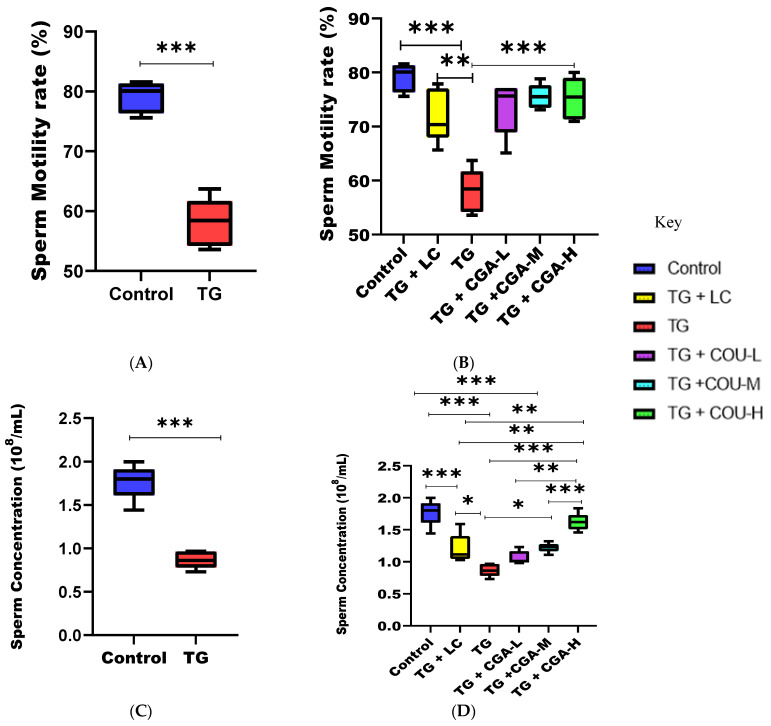
Efficacy of chlorogenic acid (CGA) on sperm quality after 28 days of experimental trials. (**A**) Effect of TG on sperm motility rate. Compared to the control, TG significantly reduced sperm motility (*p* < 0.001). (**B**) CGA significantly improved sperm motility. Compared to TG, CGA at all concentrations significantly increased the reduced level of motility (*p* < 0.001). (**C**) Effect of TG on sperm concentration. Compared to the control, TG significantly reduced sperm concentration (*p* < 0.001). (**D**) CGA significantly improved sperm concentration. Compared to the TG group, the TG + CGA-M group exhibited a significant increase (*p* < 0.05), and the TG + CGA-H group showed a significant increase as well (*p* < 0.001). Levocarnitine (TG +LC) also significantly improved the quality of sperm motility (*p* < 0.01) and sperm concentration (*p* < 0.05). There was no significant difference between the TG group and the TG + CGA-L group. (* *p* < 0.05; ** *p* < 0.01; *** *p* < 0.001).

The results revealed that the intragastric administration of TG (40 mg/kg/d) for 28 days significantly reduced sperm quality, including motility and concentration, in the TG group compared to the control group (Figure 2A,B). This effect was significantly ameliorated following CGA treatment in the CGA groups compared with the TG group. There was no significant difference between the CGA group across all levels of concentrations and the TG + LC group in terms of sperm motility, as even at low concentrations, the toxicity of TG against sperm motility was significantly attenuated. However, the effect of CGA on sperm concentration was dose-dependent, as the high dosage group (TG + CGA-H) produced a better ameliorative impact on the concentration of semen compared to other treatment groups (Figure 2C,D).

### 3.3. CGA Ameliorates TG-Induced Toxicity on Serum Sex Hormone Levels and Restores Hormonal Balance

**Figure 3:**
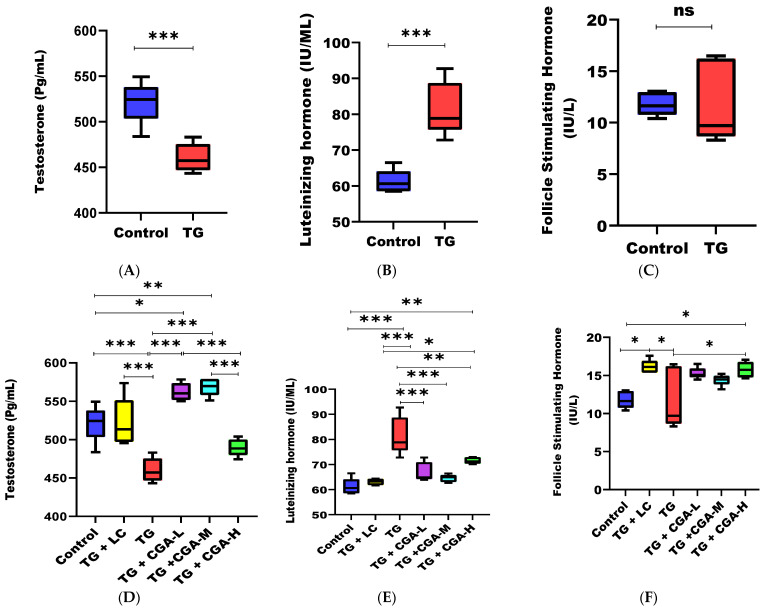
Efficacy of chlorogenic acid (CGA) on rat serum hormonal levels after 28 days of experimental trials. (**A**) Effect of TG toxicity on testosterone levels (pg/mL): Compared to the control group, TG significantly reduced testosterone levels (*p* < 0.001). (**B**) Effect of TG toxicity on luteinizing hormone (IU/mL): TG significantly increased the level of luteinizing hormone compared to the control group (*p* < 0.001). (**C**) Effect of TG on follicle-stimulating hormone (IU/L): TG increased the level of serum follicle-stimulating hormone; however, this effect was not significant. (**D**) Effect of CGA on testosterone in rats: CGA significantly increased testosterone levels in the testis. Compared with the TG group, CGA (TG + CGA-L & TG + CGA-M) significantly elevated testosterone levels (*p* < 0.001). (**E**) Effect of CGA on luteinizing hormone (IU/mL): CGA significantly reversed the toxicity of TG on luteinizing hormone levels. Compared with the TG group, TG + CGA-L and TG + CGA-M significantly reduced the elevated levels of LH (*p* < 0.001), while TG + CGA-H also showed a trend toward reduction (*p* < 0.01). (**F**) Effect of CGA on FSH levels: CGA (TG + CGA-H) significantly improved (*p* < 0.05) the effects of TG on FSH levels in rats compared to the TG and control groups. (* *p* < 0.05; ** *p* < 0.01; *** *p* < 0.001), *ns*: not significant. Colour legend is same as Figure 1 and Figure 2.

For serum hormone levels, TG significantly reduced testosterone (T) and increased luteinizing hormone (LH) levels in the TG group compared to the control group. Regarding follicle-stimulating hormone (FSH), there was an observable toxic effect of TG; however, this toxicity did not significantly alter FSH levels compared with the control (Figure 3A–C). CGA treatment significantly abrogated the toxic effects of TG on sex hormone levels, restoring T and LH to their physiological ranges at low and medium concentrations (TG + CGA-L & TG + CGA-M). While trends suggest that TG might negatively impact FSH, as indicated by the lower median level in the TG group compared to the control, both LC and CGA (TG + LC & TG + CGA-H) significantly improved the effects of TG on FSH levels in rats (Figure 3D–F). However, these findings warrant cautious interpretation and further investigation to confirm their validity.

### 3.4. CGA Improves Antioxidant Capacity and Ameliorates Oxidative Imbalance in Rat Testes

**Figure 4:**
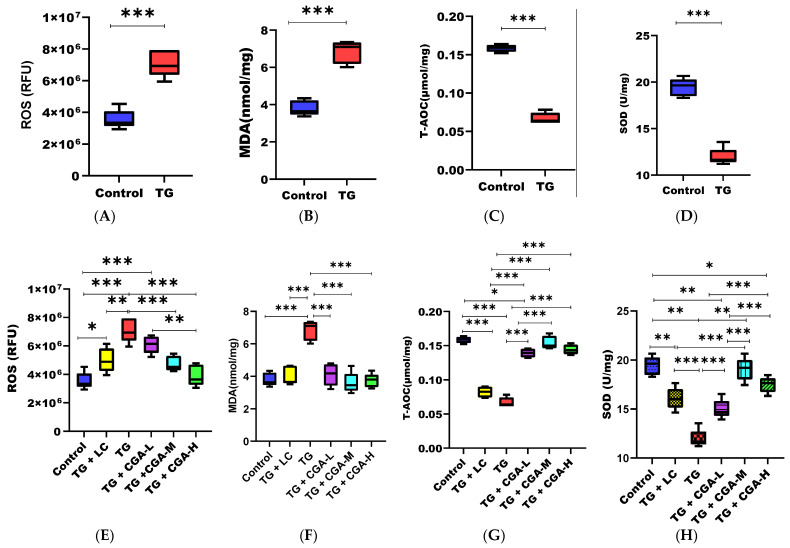
Efficacy of chlorogenic acid (CGA) on rat epidydimal and testicular tissue antioxidant status after 28 days of experimental trials. (**A**) Reactive oxygen species (ROS) (RFU). (**B**) Malondialdehyde (MDA) level (nmol/mg). (**C**) Total antioxidant capacity (T-AOC) (µmol/mg). (**D**) Superoxide dismutase (SOD) (U/mg). CGA treatment significantly reduced testicular (**E**) ROS (TG vs. TG + CGA-M & TG vs. TG + CGA-H, *p* < 0.01 & *p* < 0.001) and (**F**) MDA levels (TG vs. TG + CGA-L, TG vs. TG + CGA-M & TG vs. TG + CGA-H, *p* < 0.001) in a dose-dependent manner. Furthermore, CGA significantly increased testicular (**G**) T-AOC (TG vs. TG + CGA-L, TG vs. TG + CGA-M & TG vs. TG + CGA-H, *p* < 0.001; TG + LC vs. TG + CGA-L, TG + LC vs. TG + CGA-M & TG + LC vs. TG + CGA-H, *p* < 0.001) and (**H**) SOD levels [TG vs. TG + CGA-L (*p* < 0.01), TG vs. TG + CGA-M & TG vs. TG + CGA-H, (*p* < 0.001); TG + LC vs. TG + CGA-L, TG + LC vs. TG + CGA-M & TG + LC vs. TG + CGA-H (*p* < 0.001)]. Additionally, LC and CGA significantly improved the antioxidant status of rats in the control group. (* *p* < 0.05; ** *p* < 0.01; *** *p* < 0.001). Colour legend is same as Figure 1 and Figure 2.

To assess testicular antioxidant capacity, we measured total antioxidant capacity (T-AOC), malondialdehyde (MDA), epididymal reactive oxygen species (ROS), and superoxide dismutase (SOD) as markers of oxidative stress. Compared to the control group, the TG group exhibited significantly increased levels of MDA and ROS, along with significantly decreased levels of T-AOC and SOD (Figure 4A–D). However, treatment with CGA significantly improved the antioxidant status of the tissues, leading to increased levels of T-AOC and SOD and a concomitant decrease in MDA and ROS levels. These results indicate that CGA ameliorated oxidative stress by restoring the balance between oxidation and the antioxidant status of the testicular and epididymal tissues of treated rats (Figure 4E–H).

### 3.5. CGA Mitigates Renal and Hepatic Organs Damage in Rats

To monitor the protective effects of CGA on the liver and kidneys of experimental rats, we measured the levels of liver alanine aminotransferase (ALT) and aspartate aminotransferase (AST) as well as kidney blood urea nitrogen (BUN) and creatinine (CRE). In the TG group, ALT, BUN, and CRE levels were significantly increased compared to the control group, although AST levels did not show significant differences among the treatment groups (Table 1). The elevated levels of ALT, BUN, and CRE in the TG group indicate liver injury and impaired kidney function.

However, CGA treatment significantly reduced ALT, BUN, and CRE compared to the TG group, indicating the potential repair of kidney and liver functions. Overall, CGA treatment at moderate and high concentrations (TG + CGA-M and TG + CGA-H groups) provided better protection for liver and kidney organs, suggesting that CGA’s protective effects may be dose-dependent

### 3.6. Possible Mechanisms Using Network Pharmacology

We conducted a network pharmacology analysis to explore the mechanisms underlying the reproductive toxicity of Tripterygium glycoside (TG) tablets and the mitigating effects of chlorogenic acid (CGA). We utilized the STRING database (version 11, https://string-db.org, accessed on the 2 October 2024) to construct a protein–protein interaction (PPI) network focusing on interactions related to target proteins in Homo sapiens. This analysis revealed insights into proteins associated with CGA-related asthenozoospermia disorders. We identified a total of 423 targets, with 322 linked to sperm disorders, 92 associated with CGA and other bioactive compounds, and 9 overlapping targets implicated in both asthenozoospermia and CGA interactions were identified using Venny 2.1. The overlapping targets—AR, ESR1, HIF1A, MAPK1, NFE2L2, PTGS2, TNF, TP53, and GMNN—are crucial for reproductive health, immune response, inflammation, and cancer prevention (Figure S1A).

The initial PPI network comprised nine nodes and 28 edges, which yielded an average node degree of 6.22, a local clustering coefficient of 0.889, an expected number of edges of 15, and a PPI enrichment *p*-value of 0.00269. After excluding the empty GMNN node, a subsequent analysis revealed a functional PPI network with eight nodes, also containing 28 edges. This network had an average node degree of seven, a local clustering coefficient of one, an expected number of edges of 15, and a significant *p*-value of 0.000587, highlighting several disease-related pathways.

Using K-means clustering, we categorized the network into three clusters: one major cluster containing six genes and two minor clusters with one gene each. The major clusters comprised AR, ESR1, HIF1A, PTGS2, MAPK1, and TP53, while NFE2L2 and TNF were assigned to the minor clusters. The PPI network was visualized using Cytoscape 3.10 (Figure S1B–D). CytoHubba calculated the degree centrality (DC) for the top hub genes, and various topological parameters—such as Betweenness (BC), Closeness (CC), Eigenvector (EC), Local Average Connectivity (LAC), Network Connectivity (NC), Subgraph Connectivity (SC), and information (IC)—were computed using CytoNCA (Table S2). The results indicated that AR, ESR1, HIF1A, PTGS2, MAPK1, TP53, NFE2L2, and TNF are likely targets of CGA in mediating asthenozoospermia. Notably, the high subgraph values of AR in Table S2 emphasizes its significant interconnectivity, suggesting a central role in the molecular dynamics of spermatogenesis [61].

We subjected the six selected genes from the major cluster to Gene Ontology (GO) and Kyoto Encyclopedia of Genes and Genomes (KEGG) enrichment analyses (http://www.geneontology.org/, accessed on the 2 October 2024) to map the signaling pathways involved in asthenozoospermia. These analyses focused on biological processes (BP), cellular components (CC), and molecular functions (MF), suggesting that TG may induce reproductive toxicity through various GO terms. Specifically, TG appears to impair sperm concentration and motility by disrupting ciliary movement, axoneme assembly, and the organization of flagellated sperm. Other affected processes include the assembly of axonemal dynein complexes, microtubule-based movements, and the formation of microtubule bundles (Figure S2A,B).

Additionally, our KEGG pathway analysis identified 60 significant pathways, primarily cancer-related. However, we focused on two pathways linked specifically to spermatogenesis: the PI3K/AKT and MAPK pathways, chosen for their crucial roles in mediating spermatogenesis, cell cycle regulation, and apoptosis. These pathways elucidate the molecular mechanisms of cellular life cycles and their significance in regulating protein activities, particularly under oxidative stress conditions associated with asthenozoospermia. Elevated levels of oxidative stress can activate the MAPK cascade, including ERK1/2 (MAPK3/1), promoting apoptosis via the MAPK/PI3K/AKT signaling pathway and regulating various cellular processes (Figure 3A,B).

Thus, the PI3K/AKT and MAPK signaling pathways may serve as potential targets for the toxic effects of tripterygium glycosides as well as for therapeutic interventions involving chlorogenic acid. This provides a foundation for further experimental investigations. Thus, these insights elucidate the mechanisms of TG’s reproductive toxicity, suggesting that CGA may counteract TG’s harmful effects within the MAPK and PI3K/AKT signaling pathways by enhancing the antioxidant capacity of testicular tissues in rats and reducing oxidative stress (Figure 3A,B).

### 3.7. CGA Upregulates the Expression of PI3K and AKT

**Figure 5:**
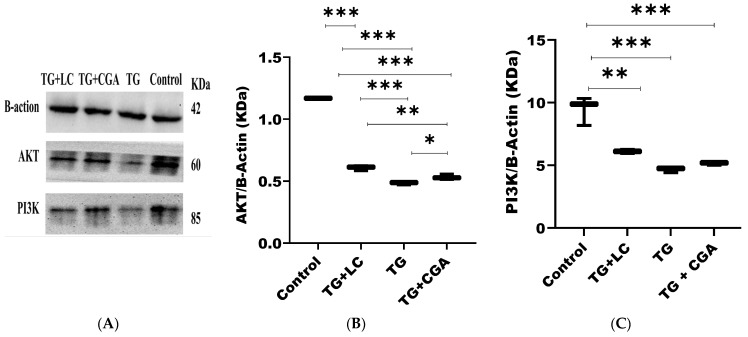
Effect of chlorogenic acid (CGA) on the expression of AKT and PI3K in experimental animals. (**A**) Chlorogenic acid (CGA) upregulated AKT and PI3K in rat testes. (**B**) CGA (50 mg/kg) upregulation of AKT was significant (*p* < 0.05). (**C**) CGA (50 mg/kg) Upregulation of PI3K was not significant compared with TG group (*p* > 0.05). (* *p* < 0.05; ** *p* < 0.01; *** *p* < 0.001).

To further elucidate the molecular mechanisms of the antioxidant action of chlorogenic acid, we measured changes in the expression of PI3K and AKT1 proteins in treated samples. It was observed that 50 mg/kg of CGA effectively attenuated the oxidative stress-induced testicular injury by upregulating the expressions of PI3K and AKT (Figure 5A–C).

### 3.8. CGA Ameliorates Intra-Testicular Hormonal Concentrations

**Figure 6:**
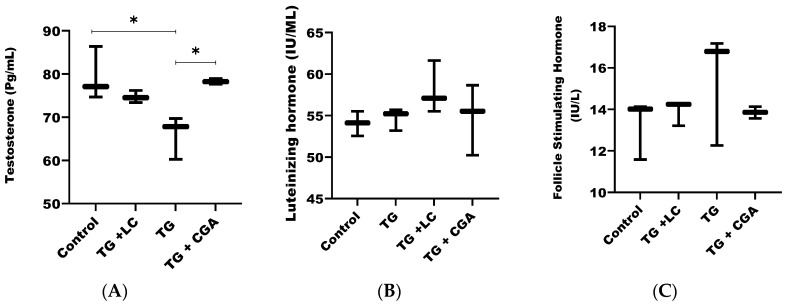
CGA ameliorates intra-testicular hormonal concentrations. (**A**) TG significantly reduced intra-testicular testosterone levels, while CGA significantly increased them (*p* < 0.05). Although TG caused notable dysregulation of intra-testicular LH and FSH, CGA resulted in a simultaneous decrease in both hormones. However, these changes were not statistically significant compared to the control (*p* > 0.05) (**B**,**C**). (* *p* < 0.05).

Having observed the effect of TG and CGA on the expression levels of PI3K and AKT, we tried to further verify whether the observed toxicity of TG and the ameliorative effect of CGA on the hormonal levels of rats were connected with the effects of TG and CGA on AKT and PI3K and thus evaluated the intra-testicular hormonal concentration of testicular samples. It was observed that TG significantly decreased the level of intra-testicular testosterone (*p* < 0.05) and increased the intra-testicular levels of the luteinizing hormone and follicle stimulating hormone. However, its increase on LH and FSH were not significantly different compared to control (*p* > 0.05). CGA (using the same concentration of 50 mg/kg) on the other hand, significantly increased the intra-testicular concentration of testosterone (*p* < 0.05). Its effect on the intra-testicular concentration of the luteinizing hormone and follicle stimulating hormone was positive but not significant (See Figure 6A–C).

### 3.9. Histopathological Analysis of Testicular Tissues

**Figure 7:**
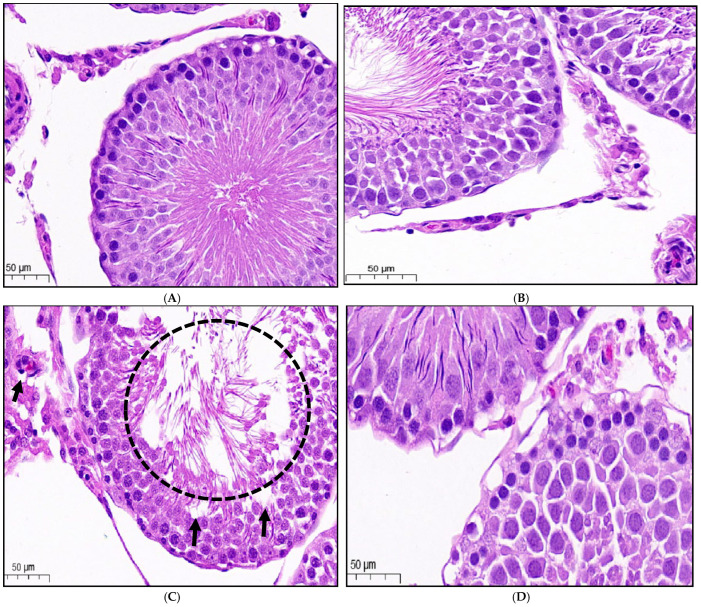

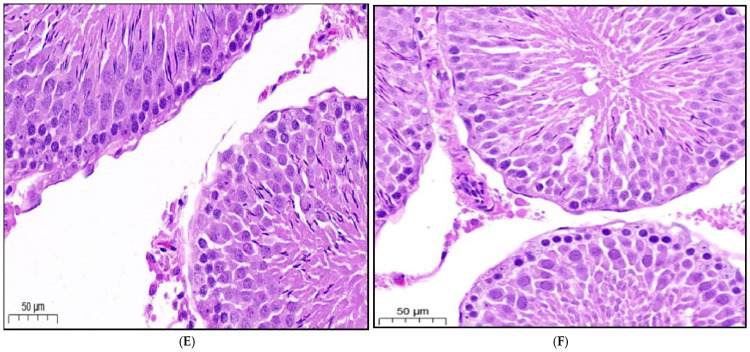
Chlorogenic acid (CGA) treatment repairs testicular damage as assessed by histopathology in male rats (H-E; 50 µm). (**A**) The control group exhibited a normal arrangement of seminiferous tubules, seminiferous epithelium, interstitial connective tissue, Leydig cells, Sertoli cells, and spermatozoa in the tubular lumen. (**B**) The TG + LC group also demonstrated a normal organization of tubules and cells. (**C**) The TG group revealed damaged tissue, characterized by disorganized and vacuolated seminiferous tubules (indicated by arrows) and enlarged intertubular spaces (broken circle), consistent with asthenozoospermia. (**D**) The TG + CGA-L group displayed partially organized and disorganized seminiferous tubules, with noticeable intertubular spaces and gaps between them. (**E**) The TG + CGA-M group showed well-organized seminiferous tubules with closed intertubular spaces and minimal gaps. (**F**) The TG + CGA-H group presented well-arranged seminiferous tubules with closed intertubular spaces and no gaps.

The histological analysis of testicular tissue in the control group showed a normal arrangement of spermatogenic cells within the seminiferous tubules, with no histopathological lesions. The TG + LC group also exhibited normal tubular and cellular organization. In contrast, the TG group displayed significant histopathological damage characterized by disorganized and vacuolated seminiferous tubules as well as enlarged intertubular spaces, indicative of asthenozoospermia. Treatment with CGA resulted in a reduction of the observed histopathological damage. The TG + CGA-H group showed the most pronounced improvement, featuring well-arranged seminiferous tubules, closed intertubular spaces, and a lack of desquamated cells (DC). The TG + CGA-L group had partially organized seminiferous tubules and pronounced intertubular spaces, while the TG + CGA-M group displayed relatively organized tubules with reduced intertubular spaces. These findings are illustrated in Figure 7A–F.

## 4. Discussion

Spermatogenesis consists of three physiological phases: (1) an initial series of mitotic divisions that primitive germ cells, or spermatogonia, undergo to form spermatocytes; (2) a second phase in which spermatocytes undergo double meiotic divisions to produce haploid spermatids; and (3) the final stage of spermatid differentiation to form functional sperm cells [62]. Any physiological change in this process or morphological damage to the organs involved can lead to spermatogenesis disorders, resulting in infertility.

In male animals or rats, particularly in cases of asthenozoospermia, infertility is characterized by reduced sperm concentration, slow sperm motility, and diminished fertilization capacity. This condition is often caused by a variety of factors, including genetic and environmental influences (such as heat, radiation, nutrition, or exposure to toxic chemicals and drugs) as well as natural phenomena (like aging and genetic mutations) that disrupt the normal progression of the three phases of spermatogenesis.

Tripterygium glycoside (TG), derived from the root extracts of Tripterygium wilfordii (Thunder God Vine), is a traditional Chinese medicine commonly used to treat rheumatoid arthritis and other autoimmune disorders. However, TG can elicit toxic effects, including hepatotoxicity, nephrotoxicity, and reproductive toxicity [63,64,65]. Our study found that TG administration resulted in weight loss, consistent with results reported by other investigators [10,66,67]. The evidence indicates that TG reduces body weight due to its high content of celastrol—a pentacyclic triterpene bioactive compound known for its anti-cancer, anti-arthritis, and anti-obesity properties. Celastrol enhances leptin sensitivity by activating the Hypothalamic Leptin Receptor-Stat 3 pathway while simultaneously reducing endoplasmic reticulum stress, thereby facilitating weight management [63,64]. However, Jiao et al. [66] reported physical changes in organs without any significant change in body weight.

In comparison to the control group, our study results showed that the intragastric administration of Tripterygium glycosides (TG) significantly reduced sperm motility and concentration, suggesting the apoptosis of sperm cells. TG disrupts normal spermatogenesis by causing testicular damage, inhibiting the proliferation and differentiation of spermatogonia, and inducing apoptosis, leading to azoospermia and oligospermia through the destruction of sperm DNA structure [64,68]. The observed reductions in body weight, sperm motility, and concentration in the TG group were attributed to TG toxicity, consistent with findings from other studies [10,63,64,65,66,67,68].

Additionally, our results demonstrated that TG increased oxidative stress levels in the testes, producing free radicals and leading to a significant rise in reactive oxygen species (ROS) and malondialdehyde (MDA) levels along with a reduction in superoxide dismutase (SOD) and total antioxidant capacity (T-AOC) [7]. Elevated oxidative stress level may have contributed to the observed injuries in liver and kidney tissues, as indicated by increased alanine aminotransferase (ALT), creatinine (CRE), and blood urea nitrogen (BUN) levels in the TG group compared to the control group [69]. This aligns with findings reported by other researchers [7,67,68,69,70].

Aitken et al. [25], Aitken and Fisher [71], Agarwal et al. [72], and Barati et al. [73] reported that oxidative stress is a primary cause of infertility in 30% to 80% of male infertile couples. This condition results from an imbalance between oxidant and antioxidant systems, often highlighted by elevated oxidative stress indicators in serum and semen and increased apoptosis compared to fertile semen. Infertile semen is typically characterized by poorly condensed sperm chromatin due to elevated ROS levels and high oxidative stress. This leads to the activation of the apoptotic cascade, resulting in a significant number of apoptotic germ cells in the testis, externalization of phosphatidylserine, increased caspase activation, and DNA damage [74].

In addition, spermatozoa are particularly susceptible to oxidative damage due to a limited array of protective enzymes, which is a consequence of their confined cytoplasmic space, and the high levels of polyunsaturated fatty acids in sperm membrane lipids also increase their vulnerability. When subjected to stressful conditions, spermatozoa activate an intrinsic apoptotic pathway, which is marked by the production of mitochondrial reactive oxygen species (ROS), a decrease in mitochondrial membrane potential, the activation of caspases, the exposure of phosphatidylserine, and oxidative DNA damage [75]. Similar results were observed in our study. TG significantly increased oxidative stress, causing kidney and liver tissue injuries, disrupted normal hormonal balance, and inhibited sperm concentration and motility, thus leading to the establishment of asthenozoospermia in male rats. This was in agreement with earlier studies [10,68]

In this study, testosterone (T) levels were significantly reduced in the TG group, while luteinizing hormone (LH) and follicle-stimulating hormone (FSH) levels increased (though not significantly), suggesting a homeostatic imbalance and dysfunction in the pituitary physiology regulating spermatogenesis due to testicular injury from oxidative stress [10,71,72,73]. The hypothalamus-pituitary-gonadal axis controls spermatogenesis and sperm maturation. Key hormones involved include the gonadotropin-releasing hormone (GnRH), secreted by the hypothalamus, and hormones produced by the pituitary gland, such as FSH, LH, and T, which is secreted by Leydig cells adjacent to the seminiferous tubules in the testes [6].

A specific threshold level of testosterone is necessary to maintain effective spermatogenesis and sperm maturation. Low testosterone levels prompt the hypothalamus to release GnRH, which binds to GnRH receptors in the pituitary gland, triggering the release of FSH and LH. Testosterone and FSH work together to stimulate spermatocyte production, growth, maturation, and the development of testicular seminiferous tubules [6].

Changes in plasma levels of FSH, LH, and testosterone (T) can be used to assess testicular tissue injury. Research has shown significant negative correlations between sperm concentration and testicular health with FSH and LH levels. Testicular damage may occur when serum FSH levels rise more sharply than LH. When testicular damage results from a significant increase in LH beyond the threshold, it is often irreversible or challenging to treat [6,76,77,78]. Tripterygium glycoside (TG) not only impairs serum levels of sex hormones but also compromises the homeostasis of intra-testicular testosterone, LH, and FSH. Strong correlations have been reported between serum testosterone and intra-testicular testosterone, as well as between serum gonadotropins and intra-testicular hormones [79]. A similar trend was observed in this study, providing evidence that the toxicity of TG on serum hormonal levels observed in this study was not coincidental.

Our findings demonstrated that TG elevates oxidative stress in the testicular environment, creating toxic conditions for the testes. This highlights the necessity of identifying antioxidant agents or drugs that can mitigate the toxic effects of TG. We investigated the impact of chlorogenic acid (CGA) on body weight, testicular tissue, sex hormone levels, testicular antioxidant status, and serum biochemical and physiological parameters and compared these effects to those of L-carnitine (LC)—a well-known dietary supplement with excellent antioxidant properties [80]. Our research utilized rat models of asthenozoospermia to explore these interactions and their implications for reproductive health.

In the TG + LC group, LC administration resulted in increased body weight compared to the TG group. However, this effect was not significantly different from that in the TG group or the other CGA treatment groups. Our results demonstrated that the TG + LC group experienced improvements in sperm motility, concentration, testosterone levels, superoxide dismutase (SOD), and total antioxidant capacity (T-AOC), while the levels of reactive oxygen species (ROS) and malondialdehyde (MDA) decreased. Additionally, reductions in serum alanine aminotransferase (ALT), aspartate aminotransferase (AST), blood urea nitrogen (BUN), and creatinine (CRE) levels were observed, although these reductions were not lower than those in the TG + CGA-M and TG + CGA-H groups. These findings suggest that L-carnitine can mitigate TG toxicity by reducing ROS and MDA production, potentially facilitating a restoration process by lowering oxidative stress [81,82]. Additionally, L-carnitine has been reported to protect against gonadal toxicity in animals exposed to high oxidative stress from toxic substances, leading to improvements in sperm count and quality due to its antioxidant properties [83].

Our study found that CGA treatment positively impacted weight management in experimental rats affected by TG administration. Similar improvements in body weight have been reported in obese rats [84,85]. Although body weights in the CGA treatment groups increased progressively with higher CGA concentrations, these weights reached physiological levels similar to those of the control group but showed no statistically significant difference compared to the TG group. This lack of significant difference was evident between the body weights of rats in the TG group and those in the TG + CGA-L, TG + CGA-M, and TG + CGA-H groups.

CGA treatment also significantly improved sperm quality by enhancing sperm motility and concentration compared to the TG group. These findings align with reports by Namula et al. [86], who demonstrated that high concentrations of CGA significantly increased sperm motility and plasma membrane integrity. Jia et al. [87] found that the oral administration of CGA improved sperm viability and enhanced acrosome and plasma membrane integrity. In another study, Pereira et al. [88] noted that CGA could improve the quality of boar semen stored at 15 °C. Specifically, our study indicated that CGA significantly enhanced sperm motility and concentration at low doses.

Additionally, Mohammadi and Hosseinchi Gharehaghaji [89] reported that semen extenders treated with rutin and CGA significantly improved the kinematic properties of sperm after thawing, particularly in terms of progressive and total motility. Anvari et al. [90] found that CGA enhanced sperm parameters, improved DNA integrity, and reduced apoptosis in mice with epilepsy.

Our study revealed that chlorogenic acid (CGA) alleviated the hormonal dysfunction caused by Tripterygium glycoside (TG) toxicity in rats. We observed a significant increase in testosterone levels and a significant reduction in the elevated levels of follicle-stimulating hormone (FSH) and luteinizing hormone (LH), thereby correcting the hormonal imbalance seen in the TG group. A similar chemoprotective effect was reported by Owumi et al. [76]. Given CGA’s chemoprotective activity in ameliorating hormonal imbalance, we assessed its effect on the antioxidant status of the rats’ testicular tissues. Our findings showed that CGA mitigated oxidative stress induced by TG, correcting the imbalance in the oxidative system by increasing the total antioxidant capacity (T-AOC) and superoxide dismutase (SOD) levels, while concomitantly decreasing malondialdehyde (MDA) and reactive oxygen species (ROS) levels. These results align with those reported by other researchers [69,76]. As a result, the antioxidant and ROS-scavenging activities of CGA reduced TG-induced toxicity to the livers and kidneys of the rats by inhibiting the production of blood urea nitrogen (BUN), creatinine (CRE), alanine aminotransferase (ALT), and aspartate aminotransferase (AST). This led to the repair of damaged livers and kidneys, restoring their function.

The findings of this study underscore the toxicity associated with the continuous use of Tripterygium glycoside [7,64] and highlight the protective properties of chlorogenic acid through various antioxidant mechanisms, as corroborated by previous research [69,70,84,85,86,87,88,89,90,91,92].

In this study, a network pharmacology analysis was used to hypothesize the mechanisms by which chlorogenic acid (CGA) mitigated the toxicity of Tripterygium glycoside (TG), utilizing relevant databases (Table S1A,B). The analysis identified 423 targets, including 322 genes associated with sperm disorders and 92 genes linked to the bioactive compounds under investigation. Among these, nine genes—AR, ESR1, HIF1A, GMNN, MAPK1/ERK2, NFE2L2, PTGS2, TNF, and TP53 (or TRP53/p53)—were identified as hub genes relevant to sperm asthenozoospermia and CGA in male rats.

The protein–protein interaction (PPI) network analysis using the STRING network builder revealed that GMNN was not directly involved in the asthenozoospermia (AZS) network. The K-means cluster analysis indicated that six genes—AR, ESR1, HIF1A, MAPK1, PTGS2, and TP53—were directly involved in sperm dysregulation in AZS, with AR having the highest subgraph value.

The proteins displayed similar values across most topological measures, including Betweenness Centrality (BC), Closeness Centrality (CC), Eigenvector Centrality (EC), Local Average Connectivity (LAC), Network Centrality (NC), Subgraph Centrality (SC), and Information Centrality (IC). AR exhibited slightly higher values in Eigenvector Centrality and Subgraph Centrality, indicating its significant interconnectivity within the network (Table S2). This underscores AR’s central role in the molecular framework of the sub-cluster, significantly influencing network dynamics [61,93,94], and emphasizes the importance of AR and other nodes in regulating spermatogenesis, motility, concentration, and capacitation.

The selected genes underwent functional Gene Ontology (GO) analysis (http://www.geneontology.org/, accessed on the 2 October 2024) to map the signaling pathways involved in the asthenozoospermia model. The GO analysis indicated that these genes regulated sperm concentration and motility through ciliary movement, axoneme assembly, cilium organization, flagellated sperm motility, and microtubule-based movement. These genes were predominantly located in the axoneme and ciliary regions of motile cilia, facilitating oxygen transport by binding to tetrapyrroles, heme, and chaperone proteins.

Additionally, the KEGG analysis indicated that these proteins mediated apoptosis in asthenozoospermia by influencing several cancer-related pathways, including thyroid cancer, bladder cancer, and central carbon metabolism. Other relevant pathways included prostate cancer, renal cell carcinoma, the VEGF signaling pathway, and additional pathways related to cell signaling and apoptosis.

Among these, the phosphoinositide 3-kinase/protein kinase (PI3K/AKT) pathway and the mitogen-activated protein kinase (MAPK) pathway are highlighted for their crucial roles in controlling apoptosis, regulating cell proliferation, and managing the cell cycle during spermatogenesis. The PI3K/AKT pathway is essential for promoting survival in various cell types [95], while the MAPK pathway is often described as a “jack of all trades” due to its ability to regulate diverse cellular functions, playing a vital role in both apoptosis and cell proliferation [96]. Together, these pathways maintain cellular homeostasis, which is critical for the proper development and function of sperm cells. Their intricate interplay underscores their importance in male reproductive health and the complex biochemistry of asthenozoospermia, highlighting the balance between promoting cell survival and regulating proliferation and differentiation during spermatogenesis [95,96,97,98].

Toxins produced by harmful substances can penetrate the testis through the blood–testis barrier (BTB) due to their small size, migrating to the testicular microenvironment, which includes Sertoli cells (SC), sperm cells, and Leydig cells (LCs). The presence of these toxins promotes the production of reactive oxygen species (ROS) [99]. Elevated ROS levels activate the MAPK pathway, including ERK1/2 (MAPK3/1) [100], which promotes apoptosis via the MAPK/PI3K/AKT signaling pathway. This apoptosis disrupts the normal structure of the testis, inhibits testosterone production, and leads to declines in sperm quality, LCs, sperm count, and motility [101,102,103,104,105].

Thus, network pharmacology studies implicated the PI3K/AKT and MAPK pathways as primary pathways activated by elevated oxidative stress due to toxic TG treatments, culminating in testicular injuries and asthenozoospermia.

To verify this network pharmacology hypothesis, testicular samples were subjected to Western blot analysis to measure the expression of PI3K and AKT proteins, as the PI3K/AKT and MAPK pathways exhibit interactions and cross-talk that support cell survival and spermatogenesis. These pathways function as reciprocal inhibitors [106]. Specifically, the RAS protein acts as a crucial connector between the MAPK and PI3K pathways, activating RAF and triggering the MAPK/ERK pathway [107], while also recruiting the p110 catalytic subunit of PI3K to the plasma membrane, thereby activating the AKT signaling pathway [108]. This relationship resembles a switch with “on/off” dynamics: activation of the MAPK pathway inhibits the PI3K/AKT pathway and vice versa, as reported by Wang et al. [109].

The Western blot analysis revealed that TG significantly inhibited PI3K and AKT protein expression in injured testes, effectively “switching on” the MAPK pathway, which led to the apoptosis of sperm cells and implicated these pathways as regulators of testicular tissue damage. Conversely, CGA increased the expression of the PI3K/AKT pathway, effectively “switching off” the MAPK pathway by reducing oxidative stress levels in treated rats. These findings underscore the potential for antioxidant interventions to mitigate toxicity and protect testicular function.

## 5. Conclusions

In conclusion, the results of this study illustrate that chlorogenic acid treatment offers protective effects against Tripterygium glycoside-induced asthenozoospermia in rats. This protective effect was evidenced by an increase in the antioxidant status of the testes, characterized by enhanced production of the superoxide dismutase (SOD) enzyme, elevated total antioxidant capacity (T-AOC) levels, and the upregulation of the PI3K and AKT proteins alongside a significant reduction in reactive oxygen species (ROS) and malondialdehyde (MDA) levels. These improvements alleviated testicular injuries and restored hormonal functions as well as serum biochemical and physiological indices, including ALT, AST, BUN, and CRE. As a result, there was a notable enhancement in sperm motility and concentration along with a restoration of renal and hepatic health, a decrease in cell apoptosis within testicular tissues, and overall improvements in spermatogenesis.

## Figures and Tables

**Table 1:** Mean ± standard deviation (SD) of biochemical and physiological indices of experimental animals in various groups.

Group	ALT ± SD (U/L)	AST ± SD(U/L)	BUN ± SD (mmol/L)	CRE ± SD (µmol/L)
**Control**	**9.50± 0.90 ^a^**	**11.83 ± 3.3 ^a^**	**8.12 ± 1.05 ^a^**	**44.41 ±3.85 ^a^**
**TG + LC**	**15.29 ± 1.19 ^b,c^**	**9.93 ± 4.54 ^a^**	**9.89 ± 2.16 ^a^**	**56.4 ± 3.62 ^a,b^**
**TG**	**22.37 ± 1.70 ^d^**	**10.28 ± 4.1 ^a^**	**20.88 ±2.29 ^c^**	**135.58 ± 11.08 ^d^**
**TG + CGA-L**	**16.48 ± 1.29 ^c^**	**10.54 ± 5.5 ^a^**	**16.15 ± 1.93 ^b^**	**73.76 ± 4.75 ^c^**
**TG + CGA-M**	**13.74 ± 0.81 ^b^**	**8.93 ± 2.5 ^a^**	**10.42 ± 1.59 ^a^**	**61.55 ± 5.95 ^b,c^**
**TG + CGA-H**	**13.4 ± 0.73 ^b^**	**11.11 ± 3.53 ^a^**	**8.38 ± 1.25 ^a^**	**55.63 ± 6.7 ^a,b^**

Reference ranges—ALT & AST: 10–40 U/L [57,58], BUN: 1.785–7.14 mmol/L [59], and CRE: 53–97.2 µmol/L [60]. **a,b,c,d**: means on the same column with different alphabets are significantly different from each other (*p* < 0.05).

## Data Availability

Additional research data, including further details on protocols, analytic methods, raw data, and processed data, will be made available upon request to interested researchers.

## Supplementary Materials

The following supporting information can be downloaded at: https://www.mdpi.com/article/10.3390/vetsci12010066/s1, Figure S1A: Venn diagram showing prediction of target genes of asthenozoospermia; Figure S1B: String prediction of nine common target genes of the asthenozoospermia PPI network; Figure S1C: String prediction of eight hub genes of the asthenozoospermia PPI network (excluding GMNN); Figure S1D: K-means sub cluster analysis showing six significant hub genes of the asthenozoospermia PPI network; Figure S2A: Bar chart of GO enrichment analysis of biological processes, cellular components, and molecular functions of target proteins; Figure S2B: Burble chart of GOenrichment analysis of biological processes, cellular components, and molecular functions of target proteins; Figure S3A,B: The PI3K-AKT pathway and the MAPK KEGG pathway; Table S1A: Databases for network pharmacological analysis; Table S1B: Databases and software for network pharmacological analysis; Table S2: Node Table based on Cytoscape Topography Measures.

## References

[1] Lü S., Wang Q., Li G., Sun S., Guo Y., Kuang H. (2015). The treatment of rheumatoid arthritis using Chinese medicinal plants: From pharmacology to potential molecular mechanisms. J. Ethnopharmacol..

[2] Lv H., Jiang L., Zhu M., Li Y., Luo M., Jiang P., Tong S., Zhang H., Yan J. (2019). The genus Tripterygium: A phytochemistry and pharmacological review. Fitoterapia.

[3] Wang H.L., Jiang Q., Feng X.H., Zhang H.D., Ge L., Luo C.G., Gong X., Li B. (2016). *Tripterygium wilfordii* Hook F versus conventional synthetic disease-modifying anti-rheumatic drugs as monotherapy for rheumatoid arthritis: A systematic review and network meta-analysis. BMC Complement. Altern. Med..

[4] Xie Z.J., Cao W., Huang L., Xun Y.Q., Yang N., Wang P., Wang Q., Ren M.J., Li H.C., Liu Y.L. (2023). Guideline for the diagnosis and treatment of rheumatoid arthritis with integrated traditional Chinese medicine and Western medicine to increase efficiency and reduce toxicity. Tradit. Med. Res..

[5] Li J., Chen D., Suo J., Zhang Y., Wang Y., Deng Z., Zhang Q., Ma B. (2024). Triptolide induced spermatogenesis dysfunction via ferroptosis activation by promoting K63-linked GPX4 polyubiquitination in spermatocytes. Chem. Biol. Interact..

[6] Zhou S.H., Deng Y.F., Weng Z.W., Weng H.W., Liu Z.D. (2019). Traditional Chinese medicine as a remedy for male infertility: A review. World J. Men’s Health.

[7] Liang S., Yin Y., Zhang Z., Fang Y., Lu G., Li H., Yin Y., Shen M. (2024). Moxibustion Prevents Tripterygium Glycoside-Induced Oligoasthenoteratozoospermia in Rats via Reduced Oxidative Stress and Modulation of the Nrf2/HO-1 Signaling Pathway. Aging.

[8] Zhang X., Xia J., Jiang Y., Pisetsky D.S., Smolen J.S., Mu R., Dai S., Weinblatt M.E., Kvien T.K., Li J. (2024). 2023 International Consensus Guidance for the use of *Tripterygium wilfordii* Hook F in the treatment of active rheumatoid arthritis. J. Autoimmun..

[9] Lin N., Zhang Y.Q., Jiang Q., Liu W., Liu J., Huang Q.C., Wu K.Y., Tu S.H., Zhou Z.S., Chen W.H. (2021). Clinical practice guideline for tripterygium glycosides/tripterygium wilfordii tablets in the treatment of rheumatoid arthritis. Front. Pharmacol..

[10] Jing X., Cheng W., Guo S., Zou Y., Zhang T., He L. (2017). Toxic effects of Tripterygium wilfordii Hook F on the reproductive system of adolescent male rats. Biomed. Pharmacother..

[11] Setty A.R., Sigal L.H. (2005). Herbal medications commonly used in the practice of rheumatology: Mechanisms of action, efficacy, and side effects. Semin. Arthritis. Rheum..

[12] Zhao L., Hartung T., Bjerrum J.T. (2015). Metabonomics and Toxicology. Metabonomics: Methods and Protocols.

[13] Zhen Q.S., Ye X., Wei Z.J. (1995). Recent progress in research on Tripterygium: A male antifertility plant. Contraception.

[14] Fan Y.F., Xu Y., Su X.H., Liu L.L., Tian Y.G., Zhao Y., Kong X.Y., Lin N. (2020). Effect of Tripterygium Glycosides Tablets on reproductive toxicity in male rats with II type collagen induced arthritis. Zhongguo Zhong yao za zhi = Zhongguo Zhongyao Zazhi = China J. Chin. Mater. Medica.

[15] Fan Y.F., Xu Y., Su X.H., Liu L.L., Tian Y.G., Zhao Y., Kong X.Y., Lin N. (2019). Effect of Tripterygium Glycosides Tablets on reproductive toxicity in female rats with II type collagen induced arthritis. Zhongguo Zhong yao za zhi = Zhongguo Zhongyao Zazhi = China J. Chin. Mater. Medica.

[16] Milardi D., Grande G., Sacchini D., Astorri A.L., Pompa G., Giampietro A., De Marinis L., Pontecorvi A., Spagnolo A.G. (2012). Male fertility and reduction in semen parameters: A single tertiary-care center experience. Int. J. Endocrinol..

[17] Akhter A., Momen S.H.M., Fatema K., Nath S.D. (2024). Prevalence of Abnormal Semen Parameters among the Infertile Couples Seeking Infertility Treatment. Mymensingh Med. J..

[18] Rowe B.H., Bretzlaff J., Bourdon C., Bota G., Blitz S., Camargo C.A. (2016). Cochrane Airways Group. Magnesium Sulfate for Treating Exacerbations of Acute Asthma in the Emergency Department. Cochrane Database Syst. Rev..

[19] Alarcón R.D., Becker A.E., Lewis-Fernandez R., Like R.C., Desai P., Foulks E., Gonzales J., Hansen H., Kopelowicz A., Lu F.G. (2009). Issues for DSM-V: The role of culture in psychiatric diagnosis. J. Nerv. Ment. Dis..

[20] Mahfouz R., Sharma R., Thiyagarajan A., Kale V., Gupta S., Sabanegh E., Agarwal A. (2010). Semen characteristics and sperm DNA fragmentation in infertile men with low and high levels of seminal reactive oxygen species. Fertil. Steril..

[21] Desai N., Sharma R., Makker K., Sabanegh E., Agarwal A. (2009). Physiologic and pathologic levels of reactive oxygen species in neat semen of infertile men. Fertil. Steril..

[22] Liguori I., Russo G., Curcio F., Bulli G., Aran L., Della-Morte D., Gargiulo G., Testa G., Cacciatore F., Bonaduce D. (2018). Oxidative stress, aging, and diseases. Clin. Interv. Aging.

[23] Valko M., Leibfritz D., Moncol J., Cronin M.T., Mazur M., Telser J. (2007). Free radicals and antioxidants in normal physiological functions and human disease. Int. J. Biochem. Cell Biol..

[24] Aitken R.J., Baker M.A. (2006). Oxidative stress, sperm survival and fertility control. Mol. Cell. Endocrinol..

[25] Aitken R.J. (2013). Human spermatozoa: Revelations on the road to conception. F1000Prime Rep..

[26] Kumar K., Thilagavathi J., Deka D., Dada R. (2012). Unexplained early pregnancy loss: Role of paternal DNA. Indian J. Med. Res..

[27] Kodama H., Yamaguchi R., Fukuda J., Kasai H., Tanaka T. (1997). Increased oxidative deoxyribonucleic acid damage in the spermatozoa of infertile male patients. Fertil. Steril..

[28] Dorostghoal M., Kazeminejad S.R., Shahbazian N., Pourmehdi M., Jabbari A. (2017). Oxidative stress status and sperm DNA fragmentation in fertile and infertile men. Andrologia.

[29] Iommiello V.M., Albani E., Di Rosa A., Marras A., Menduni F., Morreale G., Levi S.L., Pisano B., Levi-Setti P.E. (2015). Ejaculate oxidative stress is related with sperm DNA fragmentation and round cells. Int. J. Endocrinol..

[30] Bay B., Mortensen E.L., Hvidtjørn D., Kesmodel U.S. (2013). Fertility treatment and risk of childhood and adolescent mental disorders: Register based cohort study. BMJ.

[31] Davies M.J., Moore V.M., Willson K.J., Van Essen P., Priest K., Scott H., Haan E.A., Chan A. (2012). Reproductive technologies and the risk of birth defects. N. Engl. J. Med..

[32] Dianov G.L., Souza-Pinto N., Nyaga S.G., Thybo T., Stevnsner T., Bohr V.A. (2001). Base excision repair in nuclear and mitochondrial DNA. Prog. Nucleic Acid Res. Mol. Biol..

[33] Shamsi M.B., Kumar R., Bhatt A., Bamezai R.N.K., Kumar R., Gupta N.P., Das T.K., Dada R. (2008). Mitochondrial DNA mutations in etiopathogenesis of male infertility. Indian J. Urol..

[34] Feng C.Q., Song Y.B., Zou Y.G., Mao X.M. (2008). Mutation of MTCYB and MTATP6 is associated with asthenospermia. Zhonghua Nan Ke Xue = Natl. J. Androl..

[35] Aitken R.J., Clarkson J.S., Fishel S. (1989). Generation of reactive oxygen species, lipid peroxidation, and human sperm function. Biol. Reprod..

[36] Mei Y., Pan D., Jiang Y., Zhang W., Yao X., Dai Y., Yu Y., Yao X. (2019). Target discovery of chlorogenic acid derivatives from the flower buds of Lonicera macranthoides and their MAO B inhibitory mechanism. Fitoterapia.

[37] Zhang X., Zeng L., Sun T., Liu X., Hou J., Ma Q., Li Y., Lu Q., Chen S. (2019). Purification of chlorogenic acid from Heijingang potatoes and evaluation of its binding properties to recombinant human serum albumin. J. Chromatogr. B.

[38] Nishihara M., Osumi Y., Tanaka H. (2019). Examination of Extraction Conditions of Chlorogenic Acid and Its Content in Domestic Phellodendron amurense Leaves. Yakugaku Zasshi J. Pharm. Soc. Jpn..

[39] Qin G., Ma J., Wei W., Li J., Yue F. (2018). The enrichment of chlorogenic acid from Eucommia ulmoides leaves extract by mesoporous carbons. J. Chromatogr. B.

[40] Xia Z., Sun Y., Cai C., He Y., Nie P. (2019). Rapid determination of chlorogenic acid, luteoloside and 3, 5-o-dicaffeoylquinic acid in chrysanthemum using near-infrared spectroscopy. Sensors.

[41] Chen X., Cai W., Xia J., Yu H., Wang Q., Pang F., Zhao M. (2020). Metabolomic and transcriptomic analyses reveal that blue light promotes chlorogenic acid synthesis in strawberry. J. Agric. Food Chem..

[42] Wang X., Zeng Z., Tian Z., Sun J., Li Y., Fan X. (2019). Validation of spectrophotometric determination of chlorogenic acid in fermentation broth and fruits. Food Chem..

[43] McDougall G.J., Foito A., Dobson G., Austin C., Sungurtas J., Su S., Wang L., Feng C., Li S., Wang L. (2020). Glutathionyl-S-chlorogenic acid is present in fruit of Vaccinium species, potato tubers and apple juice. Food Chem..

[44] Zhao L., Wang D., Liu J., Yu X., Wang R., Wei Y., Wen C., Ouyang Z. (2019). Transcriptomic analysis of key genes involved in chlorogenic acid biosynthetic pathway and characterization of MaHCT from *Morus alba* L.. Protein Expr. Purif..

[45] Sanlier N., Atik A., Atik I. (2019). Consumption of green coffee and the risk of chronic diseases. Crit. Rev. Food Sci. Nutr..

[46] Banožić M., Jozinović A., Grgić J., Miličević B., Jokić S. (2021). High voltage electric discharge for recovery of chlorogenic acid from tobacco waste. Sustainability.

[47] Wang L., Pan X., Jiang L., Chu Y., Gao S., Jiang X., Zhang Y., Chen Y., Luo S., Peng C. (2022). The Biological Activity Mechanism of Chlorogenic Acid and Its Applications in Food Industry: A Review. Front. Nutr..

[48] Friboulet A., Thomas D. (2005). Systems Biology-an interdisciplinary approach. Biosens. Bioelectron..

[49] Yuan Z., Pan Y., Leng T., Chu Y., Zhang H., Ma J., Ma X. (2022). Progress and Prospects of Research Ideas and Methods in the Network Pharmacology of Traditional Chinese Medicine. J. Pharm. Pharm. Sci..

[50] Barabási A.L., Gulbahce N., Loscalzo J. (2011). Network Medicine: Network medicine: A network-based approach to human disease. Nat. Rev. Genet..

[51] Percie du Sert N., Hurst V., Ahluwalia A., Alam S., Avey M.T., Baker M., Browne W.J., Clark A., Cuthill I.C., Dirnagl U. (2020). The ARRIVE Guidelines 2.0: Updated Guidelines for Reporting Animal Research. PLoS Biol..

[52] Gao Y., Qian Q., Xun G., Zhang J., Sun S., Liu X., Liu F., Ge J., Zhang H., Fu Y. (2023). Integrated metabolomics and network analysis reveal changes in lipid metabolisms of tripterygium glycosides tablets in rats with collagen-induced arthritis. Comput. Struct. Biotechnol. J..

[53] Ma J., Sun B., Te L.G., Huang X., Zuo X., Han X.K., Wang S.S. (2024). A Dietary Supplement Jinghuosu Ameliorates Reproductive Damage Induced by Tripterygium Glycosides. Chin. J. Integr. Med..

[54] Zhang K., Ge Z., Fu L., An Q., Zhou F., Guo Y., Wang X., Lu W., Liang X., Wang S. (2018). Qilin pills alleviate oligoasthenospermia by inhibiting Bax-caspase-9 apoptosis pathway in the testes of model rats. Oncotarget.

[55] Ding Q., Wu Y., Liu W. (2021). Molecular mechanism of reproductive toxicity induced by Tripterygium Wilfordii based on network pharmacology. Medicine.

[56] Aleksander S.A., Balhoff J., Carbon S., Cherry J.M., Drabkin H.J., Ebert D., Feuermann M., Gaudet P., Harris N.L., Hill D.P. (2023). The Gene Ontology knowledgebase in 2023. Genetics.

[57] Hsu C.-Y., Lin G.-M., Chang S.-T. (2020). Hypoglycemic activity of extracts of Chamaecyparis obtusa var. formosana leaf in rats with hyperglycemia induced by high-fat diets and streptozotocin. J. Tradit. Complement. Med..

[58] Tomar A., Kaushik S., Khan S.I., Bisht K., Nag T.C., Arya D.S., Bhatia J. (2020). The dietary isoflavone daidzein mitigates oxidative stress, apoptosis, and inflammation in CDDP-induced kidney injury in rats: Impact of the MAPK signaling pathway. J. Biochem. Mol. Toxicol..

[59] Mineshita M., Tsuruda K., Pimentel R. (2020). Glutamine supplementation reduces kidney injury in rats. Nutr. J..

[60] Gharavi A.G., Landry D.W., Goldman L., Cooney K.A. (2024). Approach to the Patient with Renal Disease. Goldman-Cecil Medicine.

[61] Dehm S.M., Tindall D.J. (2007). Androgen Receptor Structural and Functional Elements: Role and Regulation in Prostate Cancer. Mol. Endocrinol..

[62] Chocu S., Calvel P., Rolland A.D., Pineau C. (2012). Spermatogenesis in mammals: Proteomic insights. Syst. Biol. Reprod. Med..

[63] Guo J., Huang Y., Lei X., Zhang H., Xiao B., Han Z., Liang C., Yang W. (2019). Reproductive Systemic Toxicity and Mechanism of Glucosides of *Tripterygium wilfordii* Hook. F. (GTW). Ann. Clin. Lab. Sci..

[64] Mele M.M., Nachvak M., Asghari-Jafarabadi M., Alipour B., Zohourtabar A., Fasihi M. (2018). The role of Tripterygium wilfordii extract in weight loss, energy expenditure, glucose and lipid metabolism. Prog. Nutr..

[65] Liu J., Lee J., Hernandez M.A.S., Mazitschek R., Ozcan U. (2015). Treatment of obesity with celastrol. Cell.

[66] Jiao W., Sun J., Zhang X., An Q., Fu L., Xu W., Xie H., Tang X., Liu J., Hu W. (2021). Improvement of Qilin pills on male reproductive function in tripterygium glycoside-induced oligoasthenospermia in rats. Andrologia.

[67] Dai Y., Sun L., Han S., Xu S., Wang L., Ding Y. (2022). Proteomic study on the reproductive toxicity of Tripterygium glycosides in rats. Front. Pharmacol..

[68] Chen W.Q., Ding C.F., Yu J., Wang C.Y., Wan L.Y., Hu H.M., Ma J.X. (2020). Wuzi Yanzong Pill–Based on Network Pharmacology and In Vivo Evidence—Protects Against Spermatogenesis Disorder via the Regulation of the Apoptosis Pathway. Front. Pharmacol.

[69] Feng Y., Yu Y.H., Wang S.T., Ren J., Camer D., Hua Y.Z., Zhang Q., Huang J., Xue D.L., Zhang X.F. (2016). Chlorogenic acid protects D-galactose-induced liver and kidney injury via antioxidation and anti-inflammation effects in mice. Pharm. Biol..

[70] Zhou X., Zhang B., Zhao X., Lin Y., Wang J., Wang X., Hu N., Wang S. (2021). Chlorogenic acid supplementation ameliorates hyperuricemia, relieves renal inflammation, and modulates intestinal homeostasis. Food Funct..

[71] Aitken J., Fisher H. (1994). Reactive oxygen species generation and human spermatozoa: The balance of benefit and risk. Bioessays.

[72] Agarwal A., Prabakaran S., Allamaneni S. (2006). What an andrologist/urologist should know about free radicals and why. Urology.

[73] Barati E., Nikzad H., Karimian M. (2020). Oxidative stress and male infertility: Current knowledge of pathophysiology and role of antioxidant therapy in disease management. Cell. Mol. Life Sci..

[74] Bisht S., Faiq M., Tolahunase M., Dada R. (2017). Oxidative stress and male infertility. Nat. Rev. Urol..

[75] Aitken R.J., Gibb Z., Baker M.A., Drevet J., Gharagozloo P. (2016). Causes and consequences of oxidative stress in spermatozoa. Reprod. Fertil. Dev..

[76] Owumi S.E., Anaikor R.A., Arunsi U.O., Adaramoye O.A., Oyelere A.K. (2021). Chlorogenic acid co-administration abates tamoxifen-mediated reproductive toxicities in male rats: An experimental approach. J. Food Biochem..

[77] Jiao S.-Y., Yang Y.-H., Chen S.-R. (2021). Molecular genetics of infertility: Loss-of-function mutations in humans and corresponding knockout/mutated mice. Hum. Reprod. Update.

[78] Smith L.B., Walker W.H. (2014). The regulation of spermatogenesis by androgens. Semin. Cell. Dev. Biol..

[79] Roth M.Y., Lin K., Amory J.K., Matsumoto A.M., Anawalt B.D., Snyder C.N., Kalhorn T.F., Bremner W.J., Page S.T. (2010). Serum LH correlates highly with intratesticular steroid levels in normal men. J. Androl..

[80] Keshani M., Alikiaii B., Babaei Z., Askari G., Heidari Z., Sharma M., Bagherniya M. (2024). The effects of L-carnitine supplementation on inflammation, oxidative stress, and clinical outcomes in critically Ill patients with sepsis: A randomized, double-blind, controlled trial. Nutr. J..

[81] Abd Elkader H.T.A.E., Hussein M.M., Mohammed N.A., Abdou H.M. (2024). The protective role of l-carnitine on oxidative stress, neurotransmitter perturbations, astrogliosis, and apoptosis induced by thiamethoxam in the brains of male rats. Naunyn-Schmiedeberg’s Arch. Pharmacol..

[82] Sawicka A.K., Hartmane D., Lipinska P., Wojtowicz E., Lysiak-Szydlowska W., Olek R.A. (2018). L-Carnitine supplementation in older women. A pilot study on aging skeletal muscle mass and function. Nutrients.

[83] Dehghani F., Hassanpour A., Poost-Pasand A., Noorafshan A., Karbalay-Doust S. (2013). Protective effects of L-carnitine and homogenized testis tissue on the testis and sperm parameters of busulfan-induced infertile male rats. Iran. J. Reprod. Med..

[84] Wang Z., Lam K.L., Hu J., Ge S., Zhou A., Zheng B., Zeng S., Lin S. (2019). Chlorogenic acid alleviates obesity and modulates gut microbiota in high-fat-fed mice. Food Sci. Nutr..

[85] Nguyen V., Taine E.G., Meng D., Cui T., Tan W. (2024). Chlorogenic Acid: A Systematic Review on the Biological Functions, Mechanistic Actions, and Therapeutic Potentials. Nutrients.

[86] Namula Z., Hirata M., Wittayarat M., Tanihara F., Thi Nguyen N., Hirano T., Nii M., Otoi T. (2018). Effects of chlorogenic acid and caffeic acid on the quality of frozen-thawed boar sperm. Reprod. Domest. Anim..

[87] Jia Z.C., Liu S.J., Chen T.F., Shi Z.Z., Li X.L., Gao Z.W., Zhang Q., Zhong C.F. (2024). Chlorogenic acid can improve spermatogenic dysfunction in rats with varicocele by regulating mitochondrial homeostasis and inhibiting the activation of NLRP3 inflammasomes by oxidative mitochondrial DNA and cGAS/STING pathway. Bioorgan. Chem..

[88] Pereira B.A., Zangeronimo M.G., Sousa R.V., Teles M.C., Mendez M.F.B., Rocha L.G.P. (2014). Effet de l’acide chlorogénique sur la peroxydation lipidique et la capacité antioxydante du sperme de verrat. Journées De La Recherhe Porcine.

[89] Mohammadi T., Hosseinchi Gharehaghaji M. (2024). The influence of rutin and chlorogenic acid on oxidative stress and in vivo fertility: Evaluation of the quality and antioxidant status of post-thaw semen from Azari water buffalo bulls. Vet. Med. Sci..

[90] Anvari M., Talebi A., Pourentezari M., Shahedi A., Rezvani M.E., Vakili M., Nezhad M.T. (2022). Effect of Chlorogenic Acid on Sperm Parameters, DNA Integrity and Malondialdehyde of Carbamazepine-Consuming Epileptic Mice. J. Pharm. Negat. Res..

[91] Tang Q., Cheng B., Dai R., Wang R. (2021). The Role of Androgen Receptor in Cross Talk Between Stromal Cells and Prostate Cancer Epithelial Cells. Front. Cell Dev. Biol..

[92] Tang Y., Fang C., Shi J., Chen H., Chen X., Yao X. (2024). Antioxidant potential of chlorogenic acid in Age-Related eye diseases. Pharmacol. Res. Perspect..

[93] Estrada E., Rodriguez-Velazquez J. (2005). Subgraph centrality in complex networks. Phys. Rev. E Stat. Nonlin. Soft. Matter. Phys..

[94] He X., Zhang J. (2006). Why do hubs tend to be essential in protein networks?. PLoS Genet..

[95] Hossini A.M., Quast A.S., Plötz M., Grauel K., Exner T., Küchler J., Stachelscheid H., Eberle J., Rabien A., Makrantonaki E. (2016). PI3K/AKT Signaling Pathway Is Essential for Survival of Induced Pluripotent Stem Cells. PLoS ONE.

[96] Sinkala M., Nkhoma P., Mulder N., Martin D.P. (2021). Integrated molecular characterisation of the MAPK pathways in human cancers reveals pharmacologically vulnerable mutations and gene dependencies. Commun. Biol..

[97] Almog T., Lazar S., Reiss N., Etkovitz N., Milch E., Rahamim N., Dobkin-Bekman M., Rotem R., Kalina M., Ramon J. (2008). Identification of extracellular signal-regulated kinase 1/2 and p38 MAPK as regulators of human sperm motility and acrosome reaction and as predictors of poor spermatozoan quality. J. Biol. Chem..

[98] Almog T., Naor Z. (2010). The role of Mitogen activated protein kinase (MAPK) in sperm functions. Mol. Cell. Endocrinol..

[99] Liu S., Tang Y., Chen B., Zhao Y., Aguilar Z.P., Tao X., Xu H. (2021). Inhibition of testosterone synthesis induced by oral TiO_2_ NPs is associated with ROS-MAPK(ERK1/2)-StAR signaling pathway in SD rat. Toxicol. Res..

[100] Han A., Zou L., Gan X., Li Y., Liu F., Chang X., Zhang X., Tian M., Li S., Su L. (2018). ROS generation and MAPKs activation contribute to the Ni-induced testosterone synthesis disturbance in rat Leydig cells. Toxicol. Lett..

[101] Hong F., Wang Y., Zhou Y., Zhang Q., Ge Y., Chen M., Hong J., Wang L. (2016). Exposure to TiO_2_ nanoparticles induces immunological dysfunction in mouse testitis. J. Agric. Food Chem..

[102] Sharafutdinova L.A., Fedorova A.M., Bashkatov S.A., Sinel’nikov K.N., Valiullin V.V. (2018). Structural and functional analysis of the spermatogenic epithelium in rats exposed to titanium dioxide nanoparticles. Bull. Exp. Biol. Med..

[103] Gao G., Ze Y., Zhao X., Sang X., Zheng L., Ze X., Gui S., Sheng L., Sun Q., Hong J. (2013). Titanium dioxide nanoparticle-induced testicular damage, spermatogenesis suppression, and gene expression alterations in male mice. J. Hazard. Mater..

[104] Jia F., Sun Z., Yan X., Zhou B., Wang J. (2014). Effect of pubertal nano-TiO_2_ exposure on testosterone synthesis and spermatogenesis in mice. Arch. Toxicol..

[105] Rommel C., Clarke B.A., Zimmermann S., Nunez L., Rossman R., Reid K., Moelling K., Yancopoulos G.D., Glass D.J. (1999). Differentiation stage-specific inhibition of the Raf-MEK-ERK pathway by Akt. Science.

[106] Li L., Zhao G.D., Shi Z., Qi L.L., Zhou L.Y., Fu Z.X. (2016). The Ras/Raf/MEK/ERK signaling pathway and its role in the occurrence and development of HCC. Oncol Lett.

[107] Pacold M.E., Suire S., Perisic O., Lara-Gonzalez S., Davis C.T., Walker E.H., Hawkins P.T., Stephens L., Eccleston J.F., Williams R.L. (2000). Crystal structure and functional analysis of Ras binding to its effector phosphoinositide 3-kinase gamma. Cell.

[108] He Y., Sun M.M., Zhang G.G., Yang J., Chen K.S., Xu W.W., Li B. (2021). Targeting PI3K/Akt Signal Transduction for Cancer Therapy. Signal Transduct. Target. Ther..

[109] Wang Z., Xie Y., Chen H., Yao J., Lv L., Li Y., Deng C., Zhang M., Sun X., Liu G. (2021). Guilingji Protects Against Spermatogenesis Dysfunction from Oxidative Stress via Regulation of MAPK and Apoptotic Signaling Pathways in Immp2l Mutant Mice. Front. Pharmacol..

