# Supplementary material for: Efficacy of Chlorogenic Acid in Treating Tripterygium Glycoside-Induced Asthenozoospermia in Rats and Its Possible Mechanisms
Source: Vet Sci. 2025 Jan 16;12(1):66. doi: 10.3390/vetsci12010066 (PMC11768533; doi:10.3390/vetsci12010066)
Supplement: Supplementary file 1 [file vetsci-12-00066-s001.zip › vetsci-3285825-supplementary.pdf]

Description of Supplementary Figures and Tables

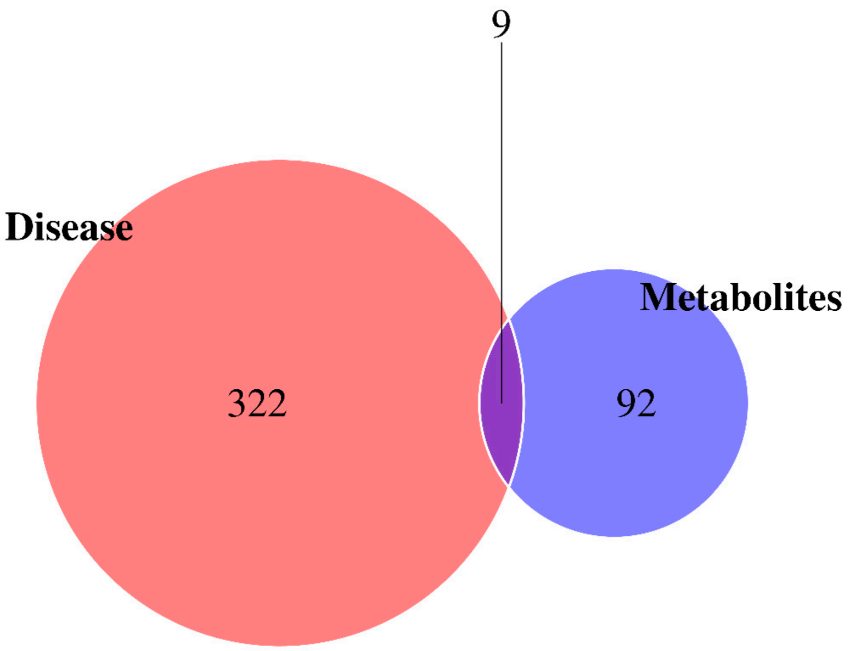

Figure. S1A. Venn diagram showing prediction of target genes of Asthenozoospermia and Metabolites (Chlorogenic Acid)

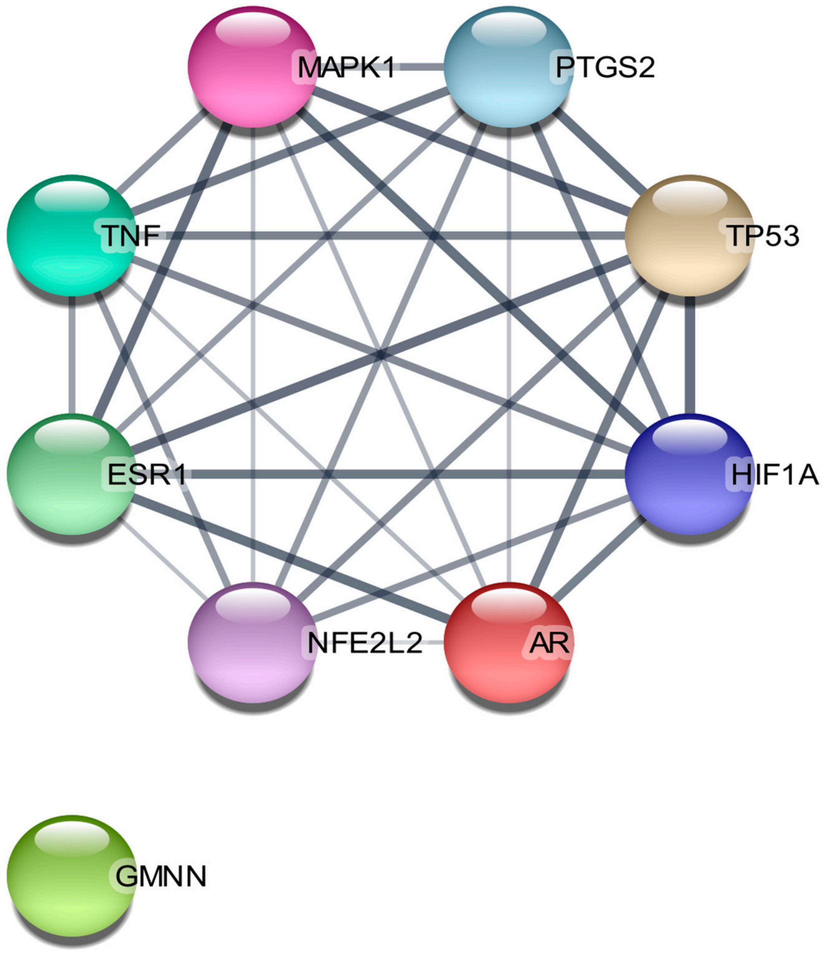

Figure. S1B. String Prediction of 9 common target genes of the Asthenozoospermia PPI-network.

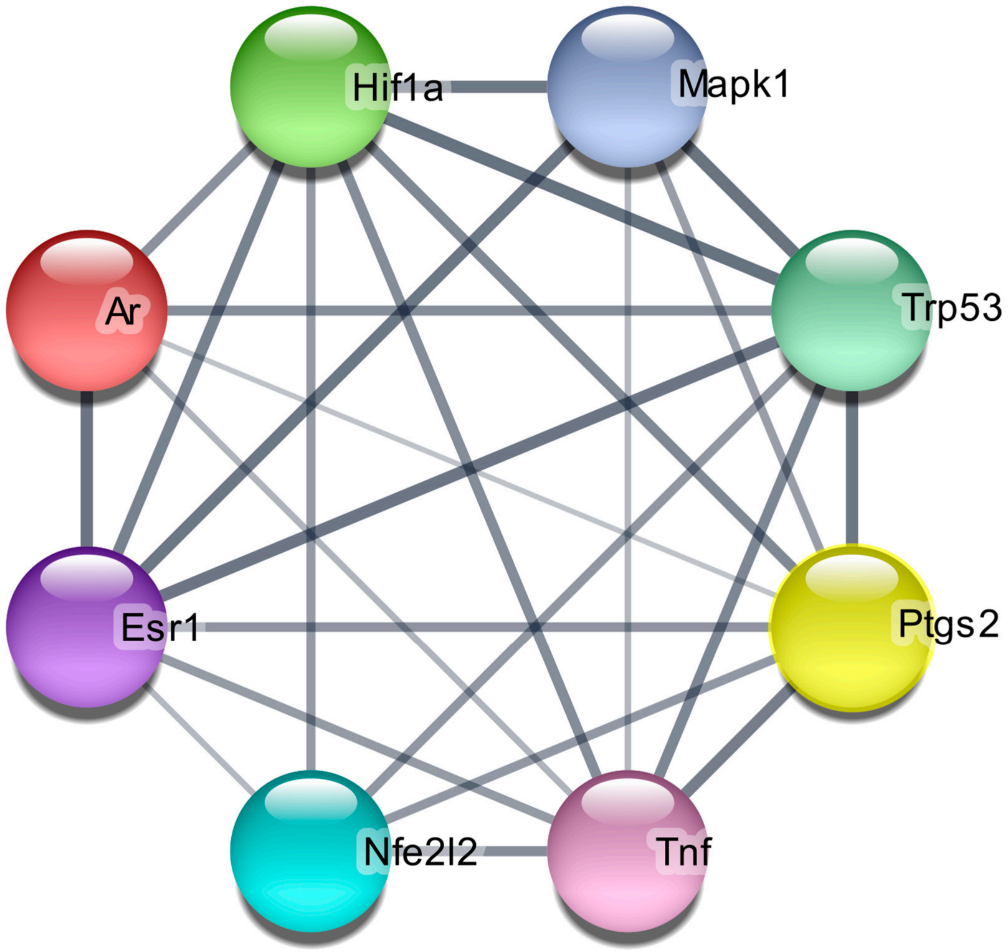

Figure. S1C. String Prediction of 8 Hub genes of the Asthenozoospermia PPI-network (Excluding GMNN).

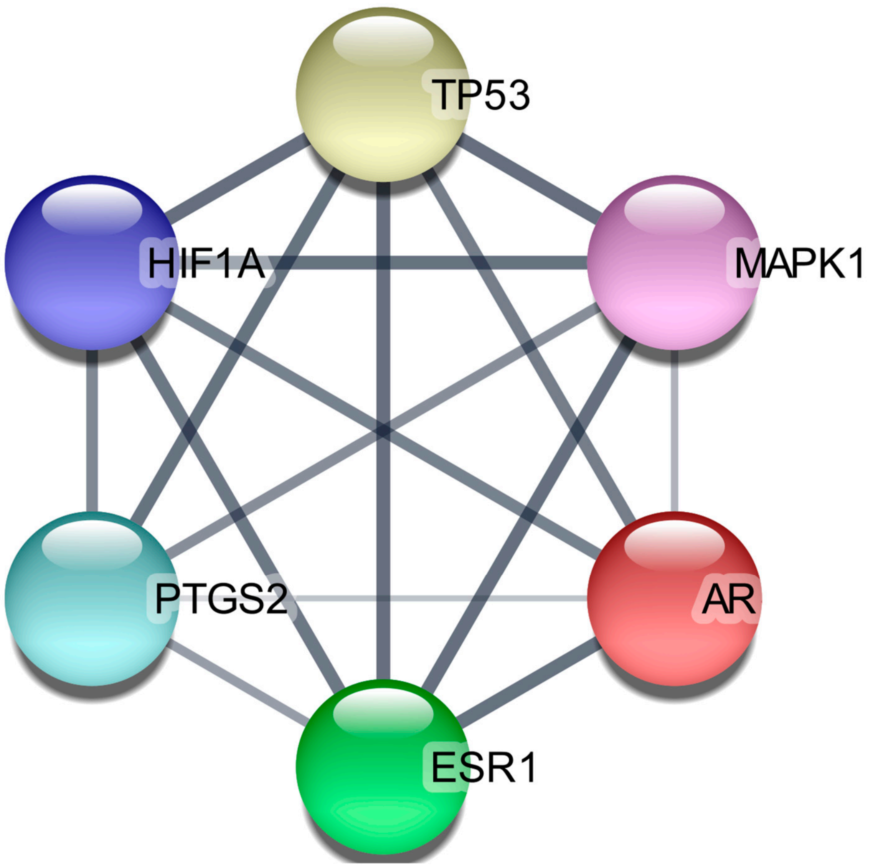

Figure. S1D. K-Means Subcluster analysis showing 6 significant Hub genes of the Asthenozoospermia PPI-network.

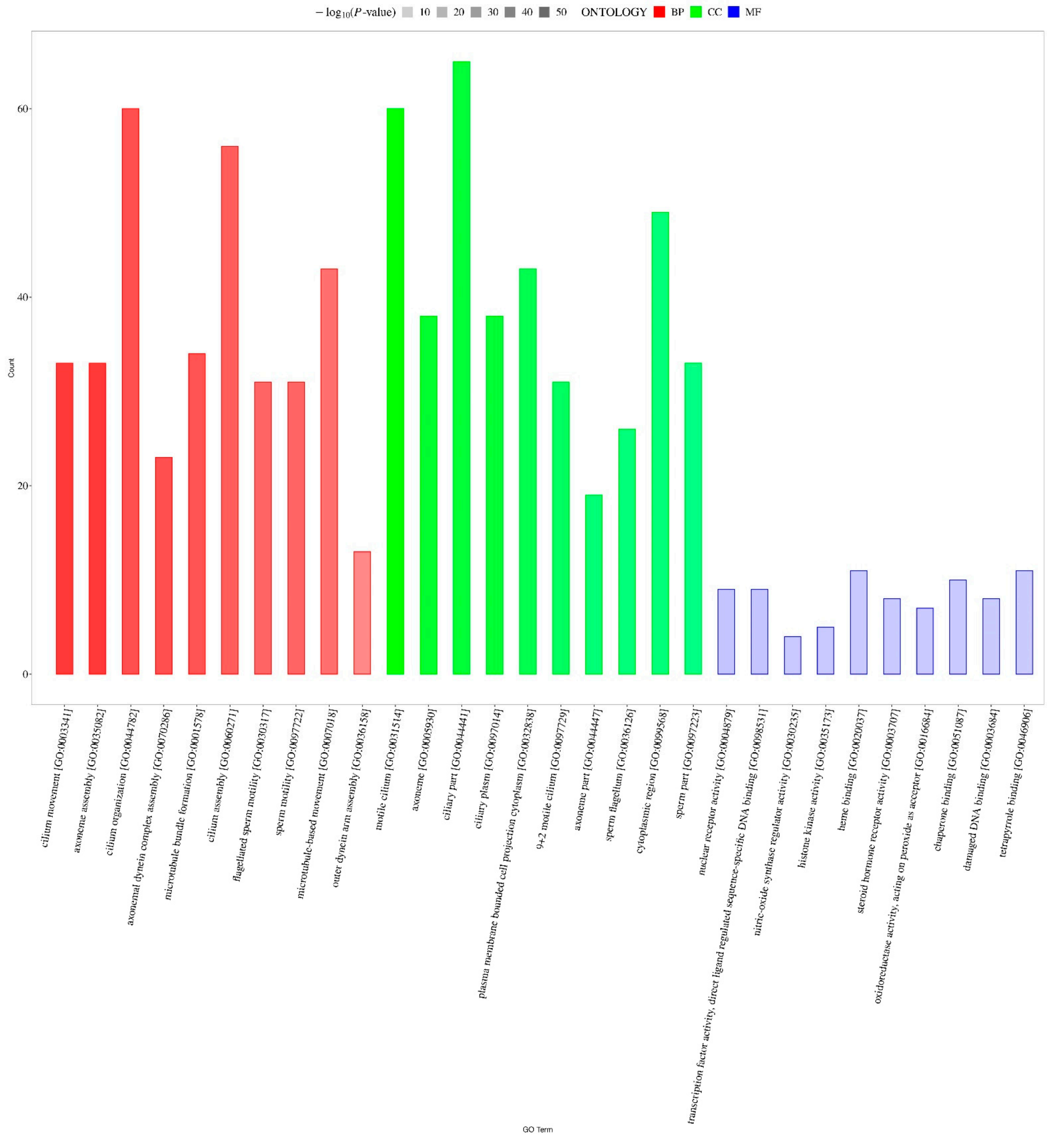

Figure. S2A. Bar chart of GO Enrichment Analysis of Biological Processes, Cellular Components and Molecular Functions of target proteins.

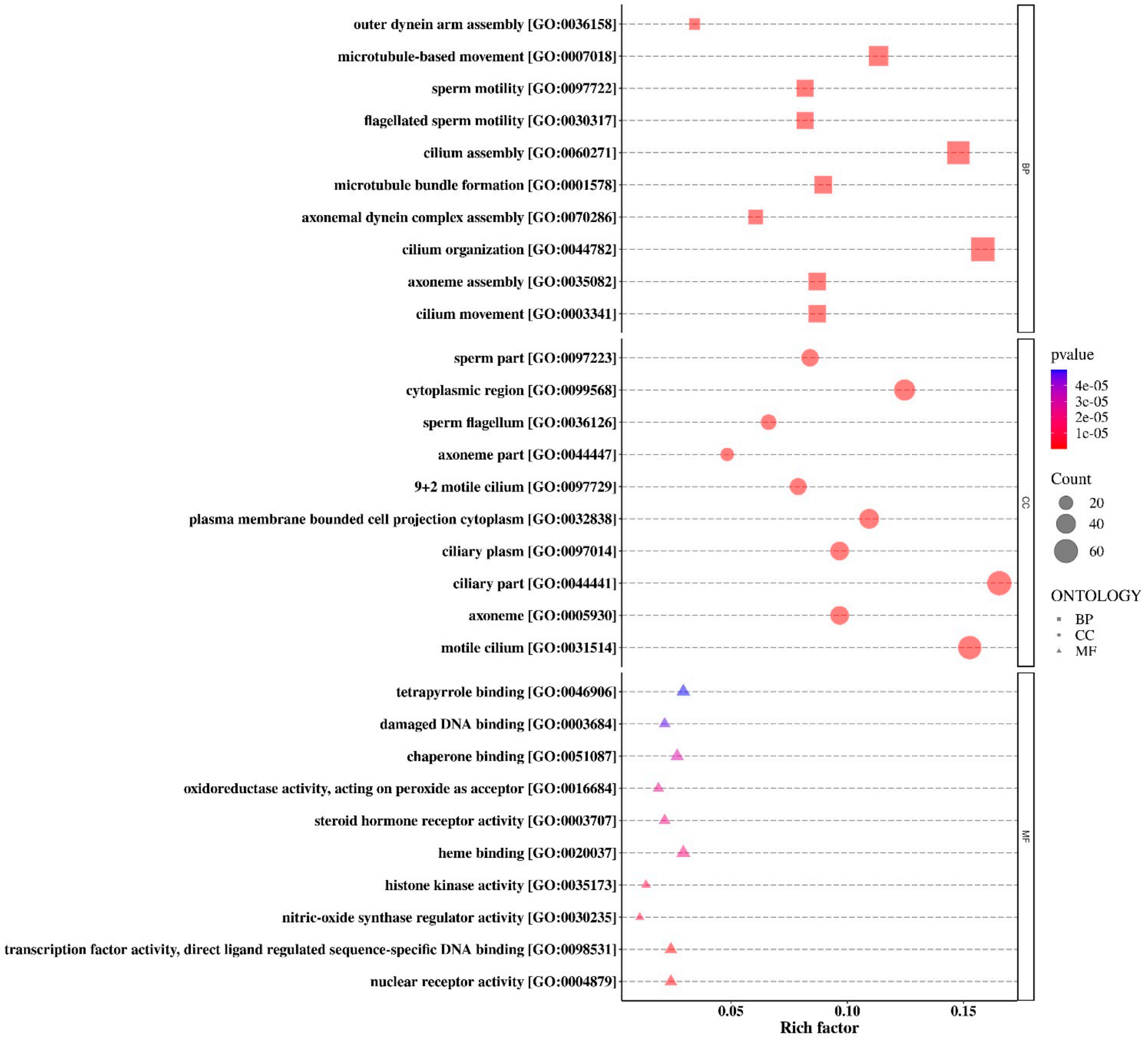

Figure. S2B. Burble chart of GO Enrichment Analysis of Biological Processes, Cellular Components and Molecular Functions of target proteins.

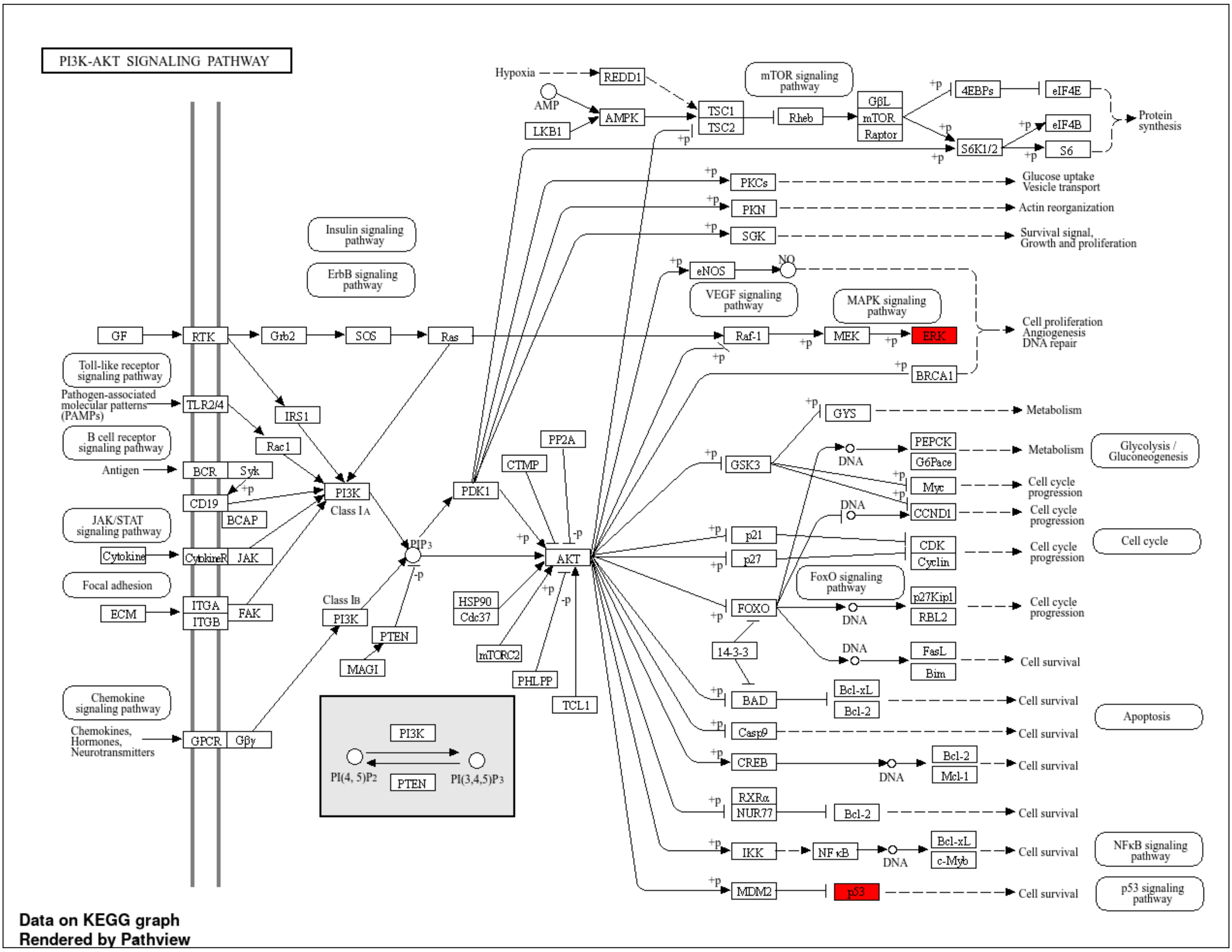

Figure S3A. The PI3K-AKT Pathway

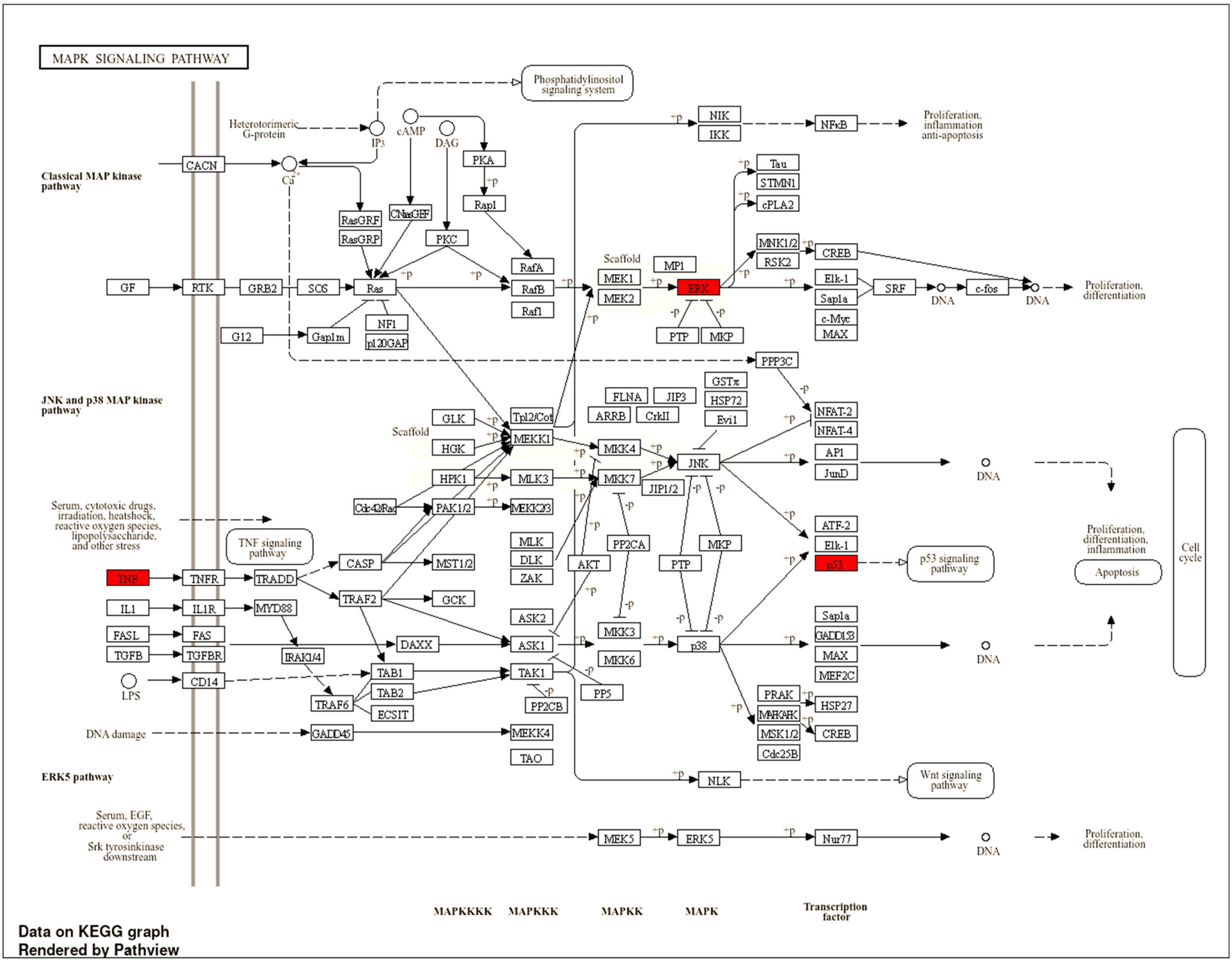

Figure S3B. The MAPK KEGG Pathway

Table S1 A: Databases for Network Pharmacological Analysis

S/N	Database	Function	Website
1	PubMed	Curating related articles	https://pubmed.ncbi.nlm.nih.gov/
2	Traditional Chinese Medicine System Pharmacopeia (TCMSP)	Identification of Bioactive Compounds from Chinese Medicine	https://old.tcmsp-e.com/load_intro.php?id=27
3	PubChem	2D structure and SMILES of Bioactive compounds	https://pubchem.ncbi.nlm.nih.gov
4	SWISS ADME	For Drug likeness of bioactive compounds	http://www.swissadme.ch/index.php
5	DrugBank	Bioactive Targets and Pathways	https://go.drugbank.com
6	UniProt	For validating the target gene ID	https://www.uniprot.org
7	DisGeNet	For disease-related genes	https://www.disgenet.org
8	GeneCards	For disease-related genes	https://www.genecards.org
9	MalaCards	MalaCards	malacards.org
10	STRING	Protein-protein interaction	https://string-db.org
11	KEGG	Gene enrichment and pathway analysis	https://www.genome.jp/kegg
12	Gene Ontology (GO)	Gene Ontology analysis	http://www.geneontology.org

Table S1B: Software for Network Pharmacological Analysis

S/N	Software	Function	Version
1	Cytoscape	Network visualization	3.10.1
2	CytoHubba	Hub genes network generation (Plugin in Cytoscape)	3.10.1
3	Microsoft Excel	Organization of Data	2024
4	Venny2.1	For drawing up the venin diagram to show common genes between bioactive and diseases	2.1

Table S2: Node Table based on Cytoscape Topography Measures

S/N	Node	Degree Centrality	Subgraph Centrality	Eigenvector Centrality	Information Centrality	LAC value	Betweenness Centrality	Closeness Centrality	Network Centrality
1	ESR1	7.0	137.40092	0.3535534	4.571429	6.0	0.0	1.0	7.0
2	HIF1A	7.0	137.40092	0.3535534	4.571429	6.0	0.0	1.0	7.0
3	TNF	7.0	137.40092	0.3535534	4.571429	6.0	0.0	1.0	7.0
4	NFE2L2	7.0	137.40092	0.3535534	4.571429	6.0	0.0	1.0	7.0
5	PTGS2	7.0	137.40092	0.3535534	4.571429	6.0	0.0	1.0	7.0
6	TP53	7.0	137.40092	0.3535534	4.571429	6.0	0.0	1.0	7.0
7	MAPK1	7.0	137.40086	0.35355332	4.571429	6.0	0.0	1.0	7.0
8	AR	7.0	137.40096	0.35355344	4.571429	6.0	0.0	1.0	7.0

LAC: Local Average Connectivity-Based Method
